# Clustered intergenic region sequences as predictors of factor H Binding Protein expression patterns and for assessing *Neisseria meningitidis* strain coverage by meningococcal vaccines

**DOI:** 10.1371/journal.pone.0197186

**Published:** 2018-05-30

**Authors:** Caroline Cayrou, Ayodeji A. Akinduko, Evgeny M. Mirkes, Jay Lucidarme, Stephen A. Clark, Luke R. Green, Helen J. Cooper, Julie Morrissey, Ray Borrow, Christopher D. Bayliss

**Affiliations:** 1 Department of Genetics and Genome Biology, University of Leicester, Leicester, United Kingdom; 2 Department of Mathematics, University of Leicester, Leicester, United Kingdom; 3 Meningococcal Reference Unit, Public Health England, Manchester Royal Infirmary, Manchester, United Kingdom; 4 School of Biosciences, University of Birmingham, Birmingham, United Kingdom; Universidad Nacional de la Plata, ARGENTINA

## Abstract

Factor H binding protein (fHbp) is a major protective antigen in 4C-MenB (Bexsero^®^) and Trumenba^®^, two serogroup B meningococcal vaccines, wherein expression level is a determinant of protection. Examination of promoter-containing intergenic region (IGR) sequences indicated that nine *fHbp* IGR alleles covered 92% of 1,032 invasive meningococcal strains with variant 1 fHbp alleles. Relative expression values for *fHbp* were determined for 79 meningococcal isolates covering ten IGR alleles by quantitative reverse transcriptase polymerase chain reaction (qRT PCR). Derivation of expression clusters of IGR sequences by linear regression identified five expression clusters with five nucleotides and one insertion showing statistically associations with differences in expression level. Sequence analysis of 273 isolates examined by the Meningococcal Antigen Typing Scheme, a sandwich ELISA, found that coverage depended on the IGR expression cluster and vaccine peptide homology combination. Specific fHbp peptide-IGR expression cluster combinations were designated as ‘at risk’ for coverage by 4C-MenB and were detected in multiple invasive meningococcal disease cases confirmed by PCR alone and occurring in partially-vaccinated infants. We conclude that sequence-based analysis of IGR sequences is informative for assessing protein expression and has utility for culture-independent assessments of strain coverage by protein-based vaccines.

## Introduction

Factor H binding protein (fHbp) is a surface-exposed lipoprotein of *Neisseria meningitidis* (Nm) that binds human factor H [1,2]. Specific variants of fHbp are either a major (4CMenB, Bexsero^®^, GSK) or the sole (Trumenba^®^, Pfizer) protective antigen of recently-licensed vaccines targeting serogroup B (MenB) strains of Nm [3,4]. Peptides of fHbp can be clustered into three (variants 1 to 3) high-order homology groups [5] with 4C-MenB containing a variant 1 fHbp peptide. Critically, fHbp expression levels vary between meningococcal strains grown under identical conditions with low expression resulting in escape of fHbp-specific bactericidal antibodies [6,7]. The fHbp expression level is, therefore, one critical determinant of vaccine coverage.

**Fig 1:**
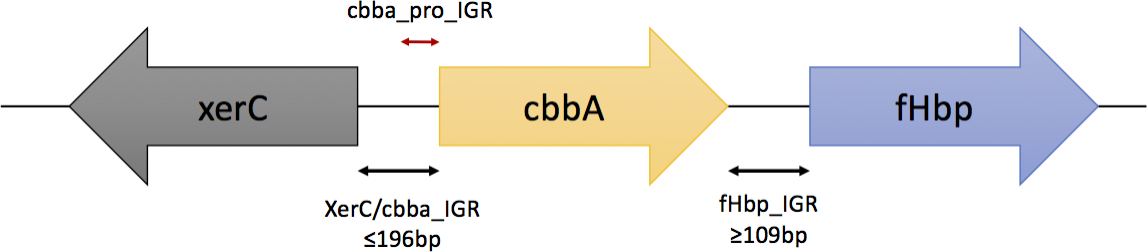
Schematic representation of the *cbbA* and *fHbp* genes and intergenic regions in genomes of *Neisseria meningitidis*. The arrows indicate the transcriptional orientation of each gene. The double black arrows mark the intergenic region (IGR) containing the promoter signal of the genes and their minimum size, the double red arrow indicates the position of the *cbbA* promoter region. The nomenclature in the PubMLST Neisseria database for these sequences is as follows: *xerC*, NEIS0351; *cbbA*, NEIS0350; *fHbp*, NEIS0349; *cbbA* IGR, igr_up_NEIS0350; *fhbp* IGR, igr_up_NEIS0349 and *cbbA* promoter, pro_NEIS0350.

The *fHbp* gene is downstream of *cbbA* (Fig 1) and this intergenic region (IGR) contains a core promoter and multiple regulatory sequences [5,8]. Expression of *fHbp* is regulated by iron availability, temperature and oxygen concentration and is also influenced in some strains by read-through transcription from *cbbA* [8–11]. Recently, Biagini *et al*. [12] determined fHbp expression levels in 105 meningococcal strains by mass spectrometry and found that particular promoter sequence clades were associated with low or high levels of fHbp expression. A minimum of 757 molecules of fHbp per bacterium were required for efficient fHbp-specific killing of meningococci in rabbit-complement SBA assays indicating that vaccines would protect against any strain expressing a homologous fHbp sub-variant at or above this level. These data covered fHbp variants from all three families and were not compared to current methods for determining strain coverage. Biagini *et al*. [12] also noted that the use of rabbit complement may have overestimated the extent of killing of heterologous fHbp variants.

Strain coverage estimates for 4C-MenB utilize the Meningococcal Antigen Typing Scheme (MATS), which assesses both homology to the vaccine variant and expression levels of fHbp in target strains [13]. The MATS threshold of protection, the Positive Bactericidal Threshold (PBT), was determined by correlation with protective serum bactericidal antibody (SBA) titres derived using pooled human sera. Application of MATS to 1,052 European MenB invasive disease isolates led to a strain coverage estimate for the fHbp component of 4C-MenB as ~65% of MenB strains [7]. A recent analysis was performed on invasive MenB meningococcal isolates obtained in epidemiological year 2014/15 from England, Wales and Northern Ireland. Variant 1 fHbp peptides were found in 68% (171/251) of these isolates but only 87% (148/171) of these variant 1-isolates were positive with MATS [14]. It was unclear if this difference was due to low expression of fHbp, low homology of these variant 1 peptides with the vaccine peptide or a combination of both phenotypes.

A major limitation of MATS for assessing 4C-MenB coverage is lack of applicability to invasive disease cases where confirmation is achieved by PCR only, without strain isolation [15]. Following introduction of 4C-MenB into the UK national infant immunisation schedule [16], development of non-culture based assays are critical for assessing coverage for the ~50% of cases confirmed by PCR alone [17]. The purpose of this study was to correlate estimates for expression of variant 1 *fHbp* alleles with *fHbp* IGR sequences and with MATS data enabling development of a sequence-based system for estimating strain coverage. This system was applied to cases of meningococcal disease in individuals immunised with 4C-MenB.

## Materials and methods

### Genome and sequence analysis

Genic and IGR sequences were obtained from the Meningococcus Genome Library (http://www.meningitis.org/research/genome; [18]) (S1 Table). The *fHbp* IGR is the sequence between *fHbp* and *cbbA* (termed igr_up_NEIS0349) while the *cbbA* IGR is the sequence between *xerC* and *cbbA* (igr_up_NEIS0350). The *cbbA* IGR contains two promoters with the *cbbA* promoter (PRO_NEIS0349) termed *cbbA*_pro_IGR herein. An fHbp homology matrix was built using peptide alignments from MUSCLE [19] and BioEdit [20] as percentage of identities against peptide 1. Putative FUR regulation sites with an nATwATnATwATnATwATn motif were detected using Fuzznuc (EMBOSS package) [21].

### *N*. *meningitidis* isolates and culture conditions

MenB invasive disease isolates and strains MC58 and H44/76 (S2 Table) were cultured either for 16–24 hrs at 37°C with 5% CO_2_ on BHI agar enriched with Levinthal’s supplement or in BHI broth at 37°C with 240 rpm shaking.

### RNA extraction and qRT PCR assays

Primers and probes for relative quantification of *fHbp* (NMB1870), *cbbA* (NMB1869) and glucose-6-phosphate dehydrogenase (*gdh*; NMB1392; the reference gene) transcripts are listed in S3 Table. Duplex and simplex amplification conditions were adjusted to obtain similar efficiencies and sensitivities for each gene (see S1 Data Set). For qRT-PCR assays, three mL of mid-log phase bacterial cultures (OD600nm of 0·5) were mixed immediately with 600 μL of 95% ethanol/5% phenol and incubated for 30 minutes at +4°C. After incubation, the bacterial cells were pelleted by centrifugation and pellet was stored at -80°C. Total RNA was extracted using a Total RNA extraction kit (Norgen, UK) as follows: addition of 100 μL of Lysozyme solution (3 mg/mL); incubation at 37°C for 10 minutes; addition of 300 μL of lysis solution with 10 μL of β-mercaptoethanol; RNA extraction following the manufacturer’s instructions; and elution in 50 μL of elution buffer. Genomic DNA was removed by two cycles of treatment for 20 minutes each using 0.5 μL of DNase solution from the Turbo DNase kit (Ambion, UK). A 500 ng sample of total RNA was subject to reverse transcription using the RNA to cDNA kit (Applied Biosystems, UK) following the manufacturer’s instructions.

Relative quantification (RQ) of expression of *fHbp* and *cbbA* involved duplex quantitative real-time PCR (qPCR) wherein *fHbp* or *cbbA* were amplified in parallel with *gdh* in triplicate. Reaction mixtures (10 μL) contained 0.5 μL of cDNA, 1X TaqMan fast advance master mix (Applied Biosystems, UK), 400 or 300 nM target gene primers, 200 nM target probe, 200 nM gdh primers and 150 nM gdh probe (S3 Table). A cDNA-free negative control and H44/76 cDNA positive control were run in parallel. Checks for contaminating DNA were performed by testing RNA samples before reverse transcription by qPCR using the same conditions. Quantification of cDNA/DNAs was performed by qPCR in an Applied Biosystems 7500HT Fast Machine using the following conditions: Uracil N-Glycosylase decontamination at 50°C for 2 minutes; polymerase activation at 95°C for 20 seconds; 40 cycles of denaturation at 95°C for 2 seconds and annealing/extension at 58°C (*fHbp*) or 60°C (*cbbA*) for 30 seconds. Ct values were determined by placing the Ct threshold in the centre of the logarithm phase. The data were validated only if a Ct value inferior to 0.3 is obtained between triplicates and if the no cDNA control exhibited a Ct value above 37. As previously described, the *gdh* gene was used to normalize qPCR data [10]. RQ values for *fHbp* and *cbbA* transcripts were determined relative to H44/76, using the 7500 Fast Applied Biosystems software and the 2^-ΔΔCt^ method [10].

### Bicistronic transcript detection by qRT-PCR and Northern blotting

Bicistronic transcripts for *cbbA/fHbp* were detected by qRT-PCR in reactions (10 μL) containing 1X Fast SYBRgreen master mix, 300 nM primers (IGF and IGR; S3 Table) and 0·5 μL of cDNA. Assay conditions were: 95°C for 20 seconds; 40 cycles of 95°C for three seconds and 60°C for 30 seconds; melt curve analysis consisting of denaturation at 95°C, renaturation at 60°C for 1 minute and progressive denaturation starting at 60°C and rising by 1% to a final temperature of 95°C.

For Northern blotting, PCR probes were produced using specific primers (S3 Table). Total RNA extracts were subject to electrophoresis in 1·2% agarose gels (0·2 μg/mL ethidium bromide, 1x MOPS, 1·1% formaldehyde) followed by transfer onto nitrocellulose membranes. Membranes were cross-linked, prehybridised in Church Gilberts buffer (0·5M disodium hydrogen phosphate, 0·5M sodium dihydrogen phosphate, 7% SDS, 1mM EDTA) and incubated with ^32^P-radiolabelled probes for 18 hours at 65°C. After four washes in 3X SSC/0·1% SDS buffer at 65°C, signal was detected by exposure on X-ray film. Relative amounts of transcripts were measured by densitometry using ImageJ software [22].

### Detection of fHbp by Western blotting

Bacterial lysates from mid-log phase cultures were separated on 12% SDS-polyacrylamide gels and transferred onto PVDF membranes. After blocking in TBST (20mM Tris, 140mM NaCl, 0·1% Tween 20)/5% skimmed milk, membranes were probed with polyclonal anti-fHbp variant 1 mouse (a kind gift of Prof. Christoph Tang) or anti-RecA rabbit antisera (Sigma, UK) and appropriate secondary antisera.

### Statistical analysis

Clusters of IGRs with a certain level of *fHbp* or *cbbA* expression (as measured by qRT-PCR) were detected by both agglomerative clustering and linear regression. For agglomerative clustering, the similarities between expression levels for each IGR were examined using the t-test and IGRs with statistically insignificant differences in mean expression levels were combined into a cluster (S2 and S3 Data Sets). These clusters satisfied two conditions: the differences between the mean qRT-PCR values for each IGR within a cluster were not statistically significant; the differences in the mean qRT-PCR values between clusters were statistically significant. For the linear regression analysis, we sought a model that predicted the average *fHbp* or *cbbA* expression level using the minimum number of variable IGR positions. The data for *fHbp* consisted of nine IGR sequences and 204 positions (i.e. nucleotides; as indicated in S1 Fig) and for *cbbA*, six IGRs and 32 positions. Each position was represented as a variable (V_n_, where n is the position in the IGR alignment) with polymorphisms coded as binary values (0 and 1; S4 Table). One *fHbp* and one *cbbA* position had three different nucleotides and these were split into two binary variables, (e.g. V_6_ and V_6_1_ for *fHbp*). Coding of all the variables produced a matrix of nine rows (IGRs) and 205 columns for *fHbp* and six rows and seven columns for *cbbA*. Removal of non-variable positions produced a matrix consisting of binary variable positions. Reiterative linear regression was applied to these remaining variables to predict the *fHbp* expression level. Individual positions whose values were statistically insignificantly different from a coefficient of zero were removed as these positions were not associated with significant differences in *fHbp* expression levels (S5 Table). This feature selection approach also identified clusters of IGRs containing variables whose coefficients did not exhibit statistically significant differences. These clusters were further examined using the t-test as described, for agglomerative clustering, and clusters whose expression values were not statistically different were merged (S6 Table and S2 Data Set). The resultant expression clusters were also examined for statistical significance by the same method (S7 Table and S2 Data Set).

A statistical analysis of MATS RP values was performed with data for four fHbp peptides. The probability of RP values, with 95% confidence intervals (CI), being below the PBT (0.012) was determined by proportion estimation assuming either a uniform or normal distribution of data (S3 Data Set).

## Results

### Diversity of *fHbp* and *cbbA* IGRs in meningococcal disease isolates

**Fig 2:**
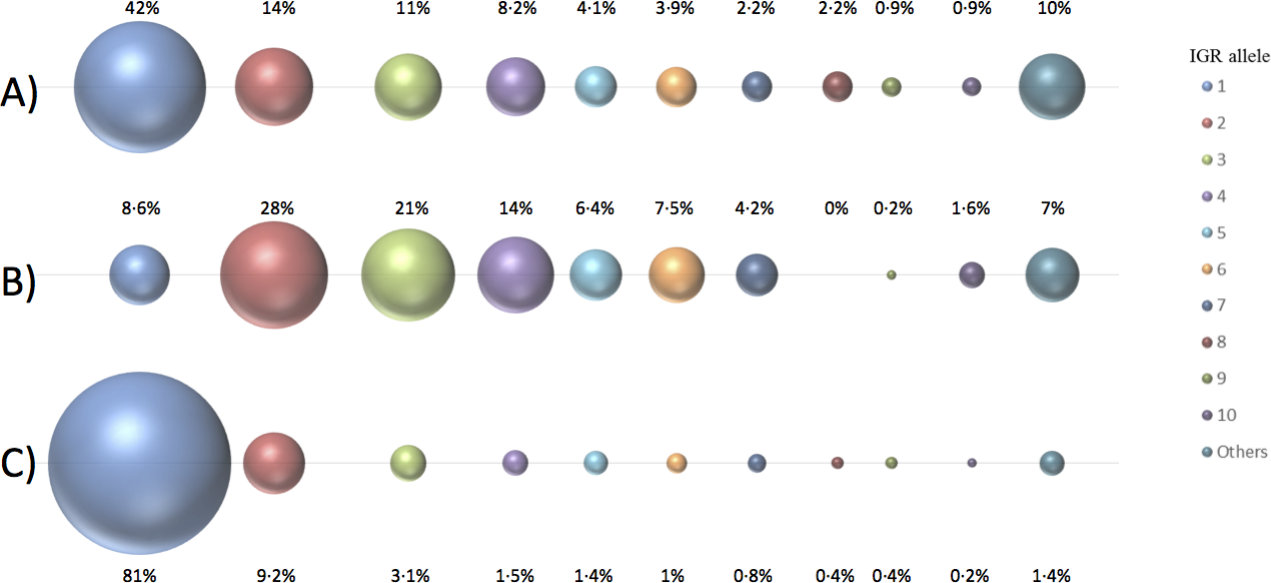
Repartition of meningococcal clinical isolates by *fHbp* and *cbbA* IGR sequence. This diagram indicates the prevalence of the promoter-containing IGR alleles of *fHbp* (S1 Fig) and *cbbA* (S3 Fig) in all the disease isolates (n = 2,008) obtained during a defined time period and from a specific geographical region. All isolates were from clinical samples that were collected in England, Wales or Northern Ireland between 2010 and 2014. The diameter of the spheres is relative to the percentage of isolates exhibiting a particular IGR sequence (actual percentages are indicated next to each sphere). The spheres represent IGR alleles 1 to 10 and all other alleles from left to right, respectively. (A) Repartition of all 2,008 isolates by *fHbp* IGR sequence allele. (B) Repartition of isolates (n = 1,032) encoding a variant 1 fHbp peptide by *fHbp* IGR sequence allele. (C) Repartition of all 2,008 isolates by *cbbA* IGR sequence allele.

A comprehensive programme of next generation sequencing and whole genome analyses of all invasive meningococcal isolates has been on-going in England, Wales and Northern Ireland since 2010 providing opportunities to robustly investigate the impact of meningococcal vaccine campaigns. We focused this study on the period between 2010 and 2014 as this period was utilized for the epidemiological studies that led to the recommendation for incorporation of 4C-MenB into the UK infant immunization schedule. Analysis of 2,008 genome sequences from all invasive disease isolates identified 110 *fHbp*_IGR sequences (S1 Fig). Only 10 different IGR sequences (fHbp_IGR_1 to 10) were present among 90% of these isolates (Fig 2A). IGRs ranged in length from 109 to 344 bp but three (56, 57 and 58) contained a ~2,000bp insertion. A sub-set of 1,032 isolates possessed fHbp variant 1 peptides of which 953 were MenB isolates. Nine IGRs covered 91.5% of this sub-set with fHbp_IGR_2 being the most frequent (Fig 2B).

Sanders *et al*. [10] observed bicistronic cbbA-fHbp transcripts and co-regulation of these genes. Examination of the *cbbA* IGR identified a core promoter within 101 bp of the *xerC* initiation codon and a breakpoint between two variable regions indicating that the *cbbA* promoter region, *cbbA*_pro_IGR, is within a 93 bp sequence (Fig 1). Analysis of 2,002 isolates identified 26 *cbbA*_pro_IGR alleles with allele 1 present in 81% of isolates (Fig 2C). The *fHbp* and *cbbA*_pro IGR alleles are available within the PubMLST *Neisseria* database [23].

### Detection and quantification of *fHbp*, *cbbA* and bicistronic transcripts by qRT PCR

In order to determine the relative levels of expression associated with *fHbp* IGRs, a subset of 79 isolates were selected that represented the commonest *fHbp* IGRs across a range of clonal complexes and a few rarer IGRs. Transcripts of *fHbp* were successfully detected and quantified by qRT PCR using *gdh* as an internal control. Relative expression of *fHbp* (versus H44/76) ranged from 0·22 to 1·61, a fold difference of 7·3 (S2 Table). Quantification of *fHbp* transcript levels by probing Northern blots of RNA extracts from 15 isolates detected a similar trend in RQ values as detected by qRT PCR (S2 Fig).

The expression levels for *cbbA* IGRs were also assessed due to the co-regulation of *cbbA* and *fHbp* in some strains. Relative expression of *cbbA*, with six different IGRs, was measured by qRT PCR and for five of the IGRs RQ values were between 0.47- and 1·55-fold as compared to H44/76 (S2 Table). For *cbbA*_pro_IGR, allele 6, the mean RQ values ranged from 0.25 to 0.57, indicating a low level of expression, however this allele was only found in 1% of strains (Fig 2).

### Associations between IGR sequence type and levels of *fHbp* and *cbbA* transcripts

**Fig 3:**
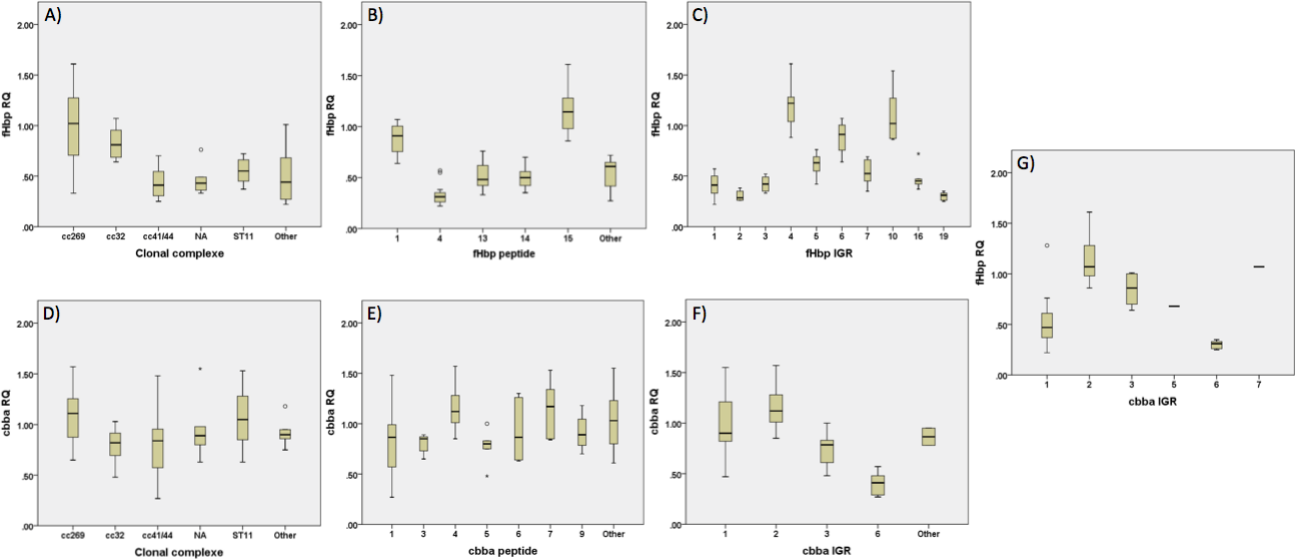
Comparison of relative expression levels for *fHbp* or *cbbA* transcripts. **Transcripts were measured by qRT PCR using *gdh* as an internal control.** Relative quantification (RQ) values were determined relative to strain H44/76 that was given an arbitrary value of 1. RQ values for *fHbp* (A, B, C, and G) and *cbbA* (D, E and F) were stratified by clonal complex (A, D), fHbp and cbbA peptides (B and E respectively) and IGR alleles (C, F, G).

The clonal structure of meningococcal populations could result in linkage of fHbp expression levels with other genetic markers. Comparison of *fHbp* RNA expression levels between lineages indicated that the cc269 and cc32 lineages have higher expression than other ccs (Fig 3A). The cc269 strains split into two groupings with low (0·33–0·55; ST-1161 and ST-5972; the ST-275 cluster) and high (0·85–1·54; ST-269 and ST-269-cluster isolates) expression. An association between *fHbp* transcript levels and fHbp peptide was also observed with peptides 1 and 15 exhibiting relatively high amounts of transcripts while peptide 4 was mainly associated with lower levels (Fig 3B). Variation in RQ values within peptide groups was low being between 1·9- and 2·6-fold. However, the strongest association was observed for *fHbp* expression and IGR sequences with these groups exhibiting an average of 1·8-fold variation in *fHbp* RQ values (Fig 3C). This association between *fHbp* expression level and IGR sequence indicates that the *fHbp* IGR is the major determinant of *fHbp* gene expression, however the weaker associations with fHbp peptide and clonal complex suggest that expression of this gene may also be linked and co-evolving with other meningococcal genetic markers. In contrast, the *cbbA* expression data was similar for all ccs and CbbA peptides (Fig 3D and 3E). This lack of a linkage effect for *cbbA* is mainly due to the low levels of variation in expression levels for this gene that is the expected pattern for a conserved house-keeping gene.

### Bicistronic *cbbA*-*fHbp* transcripts

To detect the presence of bicistronic transcripts, we performed both qRT PCR, with a probe located in the *fHbp* IGR, for 79 isolates (S2 Table), and Northern blotting (S2 Fig) for a subset of isolates. We detected combined *cbbA*-*fHbp* transcripts for multiple cc32 (n = 6), and cc269 (n = 14) isolates and one cc162 isolate, which collectively featured five combinations of fHbp_IGR and four *cbbA*_pro_IGR alleles (i.e. 4+2, 4+1, 6+7, 6+3, 10+2) but with a preponderance of PRO_NEIS0350 alleles 2 and 3 (19 of 21). In all of these cases, high RQ values were detected for *fHbp* (average, 1.06; range, 0.64–1.61; SD, 0.26) and *cbbA* (average, 1.02; range, 0.48–1.57, SD, 0.27), suggesting that read-through transcription is a major determinant of *fHbp* transcript levels (S2 Table).

### Analysis of associations between IGR variation and fHbp expression levels

**Fig 4:**
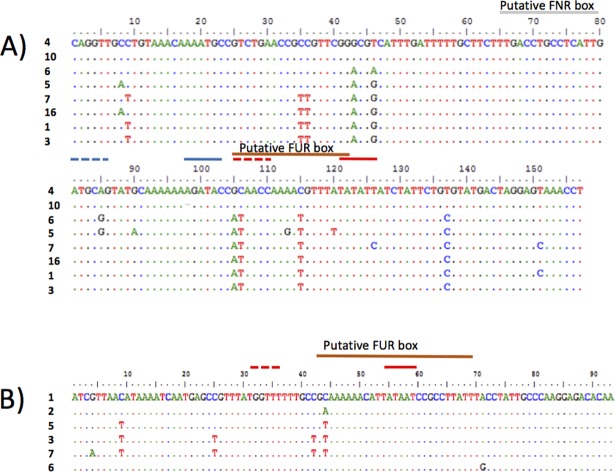
Intergenic region sequence alignment showing the positions of regulatory sequences. **Sequences are ordered from high (top) to low (bottom) expression.** (A) Alignment of seven of the most frequent fHbp IGR sequences in strains with a variant 1 fHbp. Both i2 and i19 sequences were excluded due to the presence of large insertion elements. The sequence variants associated with statistical differences in *fHbp* RQ values (see text) were:- V1, C to A at position 8; V2, C to T at position 9; V3, CC to TT at positions 35 and 36; V7, A to G at position 85. (B) Alignment of six of the most frequent *cbbA*_pro_IGR sequences. Blue and red lines indicate the positions of putative promoters, in each case the dashed and intact lines indicate the -35 and -10 boxes, respectively.

Variation in RNA expression levels is expected to correlate with allelic variation at specific positions of promoter sequences. Alignment of 106 *fHbp* IGR sequences (incomplete sequences or alleles with large insertions were excluded) identified 65 polymorphic sites and 16 insertions of 1 to 186 bp. Insertion of a 186 bp AT rich-element was associated with low *fHbp* expression (fHbp_IGR_2). Polymorphic sites were present in the putative core transcription box (i.e. -35 and -10) and Fur consensus binding site (Fig 4A, S1 Fig).

The *cbbA* promoter exhibited a similar level of variability with 30 polymorphic sites and two small insertions (i.e. 1 or 2 nucleotides; S3 Fig). However, in the six alleles analysed by qRT-PCR, and representing ~97% of 2,008 meningococcal isolates (Fig 2), there were only six polymorphic sites. Intriguingly, there was only a single polymorphism located outside of any conserved motifs between *cbbA* promoters associated with high and low expression, cbbA_pro_IGR alleles 1 and 6 respectively (Figs 3F and 4B), indicating that this polymorphism is a major determinant of expression level. The other higher expressing *cbbA* promoter alleles all contained polymorphisms in the spacer between the core promoter elements, suggesting that these polymorphisms do not impact on *cbbA* transcript levels (Fig 4B)

**Fig 5:**
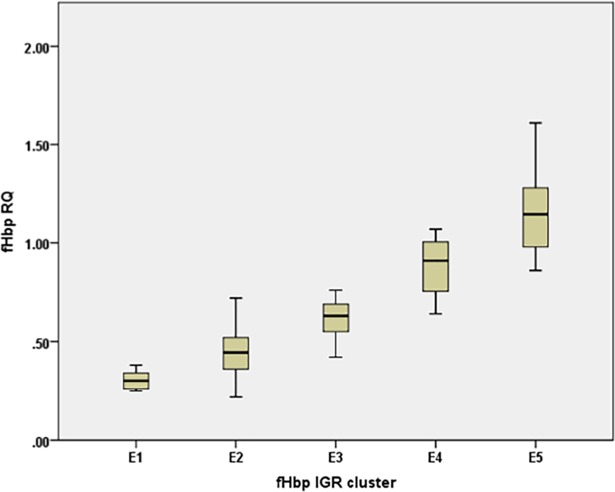
Relative expression for *fHbp* IGR expression clusters. Expression clusters (E) were derived by a linear regression analysis of the RQ values obtained for each *fHbp* IGR allele as outlined in the text. The sequences and prevalence in the 2010–2014 England, Wales and Northern Ireland meningococcal disease isolates of the *fHbp* IGR alleles are shown in S1 Fig and Fig 2, respectively. Each cluster represents the following *fHbp* IGR alleles: E1, i2 and i19; E2, i1, i3, i7 and i16; E3, i5; E4, i6; and E5, i4 and i10.

Two approaches were utilized to examine the statistical significance of differences between mean qRT PCR values of each *fHbp* IGR for the 79 isolates. Agglomerative clustering and linear regression identified six and five clusters, respectively, of IGRs with non-significant differences in their expression values with linear regression merging two agglomerative clusters into one cluster (i.e. i1 and i3 were merged with i7 and i16). Linear regression avoids overfitting of data and was adopted for on-going analyses. The five clusters identified by linear regression (E1 to E5) consisted of one to four IGRs (S7 Table, Fig 5). These clusters were based on associations between five specific combinations of polymorphisms and gene expression levels that are predictive of *fHbp* transcript levels with a goodness of fit of 81% (S4 and S5 Tables).

Allelic variation within each cluster ranged from 1–4 polymorphic sites. As expression levels of IGRs within a cluster are not significantly different, these polymorphic sites were disregarded as determinants of mRNA expression for *fHbp*. Eleven polymorphic sites were detected between the highest expression cluster (E5) and other clusters. Three of these expression-controlling polymorphism combinations (V1, V2 and V3) were present within a 36 bp region immediately downstream of the *cbbA* gene (Fig 4; S5 Table). This region is predicted to contain a stem-loop structure composed of two inverted uptake sequences (i.e. 5’GCCGTCTGAAnnnnnnTTCGGACGGC3’) followed by a five nucleotide polyU sequence, which may act as a Rho-independent terminator [8]. The IGRs associated with high expression (i.e. 4, 6 and 10) have alternate sequences for the second uptake sequence, which may disrupt the formation of the stem loop structure. Intriguingly, the statistically significant polymorphic V3 combination results in a change in two nucleotides in the four-nucleotide spacer located between these uptake sequences, suggesting that this loop may regulate hairpin formation. The other statistical significant variant nucleotides are single nucleotide differences in the putative -35 adjacent to the FNR box (V7) and a large insertion element (V15) with the latter being associated with *fHbp* IGR allele 2, which has the lowest level of expression.

### Analysis of associations between *cbbA* IGR variation and *cbbA* transcript levels

The linear agglomerative clustering and linear regression approaches were also applied to detect associations between *cbbA* IGRs and nucleotide variants with cbbA qRT-PCR values. Both approaches identified four *cbbA* IGR expression clusters (Ecb) with statistically significant differences in average qRT-PCR values. These clusters consisted of one or two IGRs: Ecb1, IGR 2; Ecb2, IGRs 1 and 5; Ecb3, IGRs 3 and 7; Ecb4, IGR 6. Four variable nucleotides (V3/V4, V5_a and V6; see Fig 4B) were associated with the differences in qRT-PCR levels between each cluster (S2 Data Set). V3/V4 consisted of two co-segregating nucleotide variants with one adjacent to the putative -35 binding site of the RNA polymerase and another in the spacer between the -10 and -35 elements. This latter position is also the location of V5_a, an A variant that converts a 6A tract to a 7A tract. The V5_a and V6 variables were also in a putative Fur binding site.

### Comparison of qRT PCR, IGR allele and MATS for fHbp

**Fig 6:**
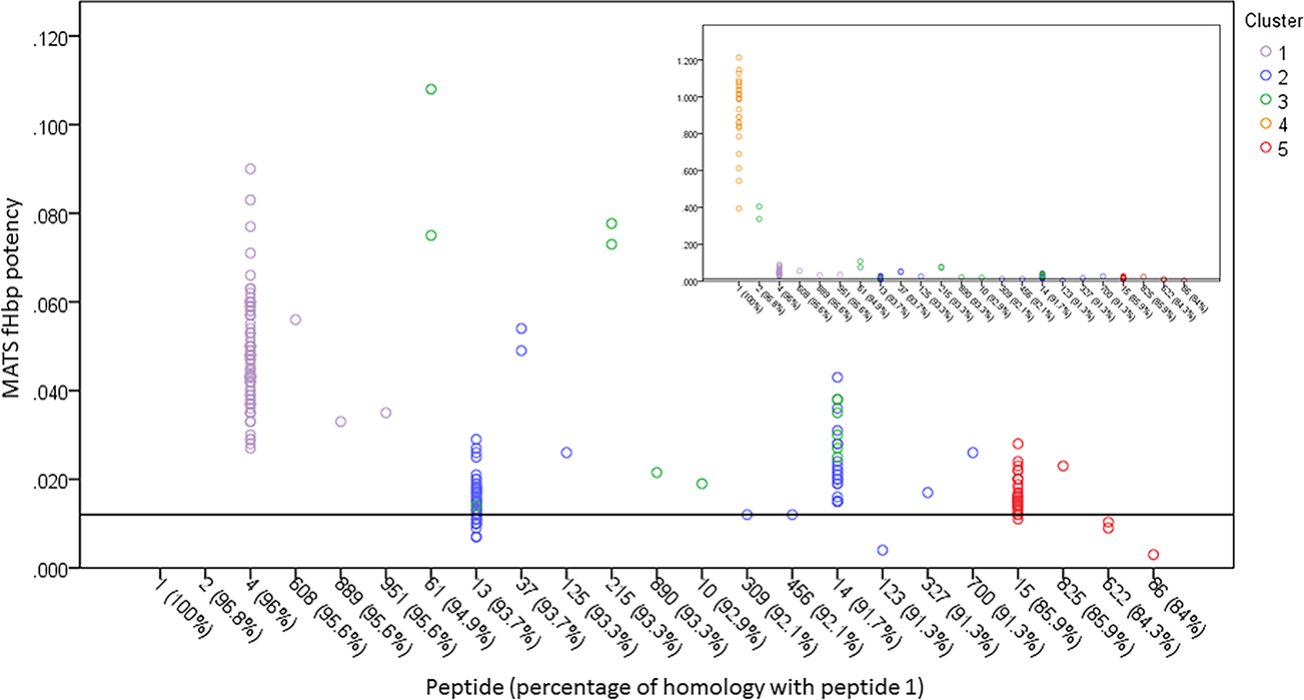
Combinatorial effects of expression level and homology to the 4C-MenB vaccine antigen for MATS-based estimates of fHbp strain coverage. All isolates (n = 132) were MenB strains with variant 1 fHbp alleles. Each symbol represents one isolate. The y-axis is the relative potency (RP) score obtained in an fHbp MATS assay. The line represents the fHbp PBT below which an isolate is defined as negative for coverage by fHbp. The x-axis is the fHbp peptide sequence and the corresponding percentage of homology observed with fHbp peptide 1.1. Expression clusters (E1 to E5) are represented by specific symbols as indicated. Samples exhibiting an RP above 0.110 are excluded from the main plot. The inset panel represents the same samples plotted with an extended y-axis range allowing inclusion of samples exhibiting RP values above 0.110.

As strain coverage for 4C-MenB is usually determined by MATS, *fHbp* IGR expression clusters were compared to MATS relative potency (RP) scores for 264 MenB isolates (S8 Table). Cluster E4 was associated with high average RP values (0.93 ±0·19) and clusters E2 and E5 (0·018 ±0.008 and 0·016 ±0.005, respectively) with low RP values while E1 and E4 had intermediate values (0.048±0.012 and 0.088±0.12). There were 32 isolates with RP scores equal to or below the PBT (i.e. 0.012) with 25 in cluster E2 and 7 in cluster E5 representing 27% and 19% of all isolates in these clusters respectively, indicating that these IGR clusters may identify isolates whose *fHbp* expression is ‘borderline’ in a MATS estimate of protection. Surprisingly cluster E5 is associated with high *fHbp* transcript levels, however these IGR types are mainly associated with fHbp peptide 15, which has only 86% identity with peptide 1. This suggests that high expression compensates for low homology with the vaccine antigen. We therefore examined the correlations between MATS, expression cluster and peptide (Fig 6). RP values equal to or below the PBT were found for several isolates containing E2 and peptide 13, for a small number with E5 and peptide 15 and six other rare combinations of peptides and E2 or E5 clusters. One of these latter combinations, isolate M04_240731 (E2/peptide 123), was investigated further by Northern and Western blotting (S2 and S4 Figs). This isolate exhibited a high level of transcript but low levels of protein, indicating that the non-synonymous point mutation (632C>T, serine to phenylalanine) present in this fHbp peptide may either reduce translation or destabilise the fHbp protein.

Our results indicated that the homology of fHbp peptide to the vaccine antigen is partially responsible for determining the MATS RP values. The fHbp peptide has previously been observed to have a modular structure with five segments (A to E) that are major determinants of peptide type [24,25]. Therefore, we examined the associations between *fHbp* expression cluster, fHbp amino acid segment and MATS RP values and we observed that two specific combinations of the C and E segments and *fHbp* IGR were associated with isolates spanning the PBT (S5 Fig). In one case, segments C1.4 and E1.4 were linked with high expression but contained isolates below the PBT. As expression is high for these isolates, this result might suggest that low homology of these segments is responsible for the low MATS value. However, these isolates contain peptide 15, which, in contrast to most other isolates, exhibits high divergence in segment A, the N-terminal domain, and that this domain may be the key determinant of the low RP values (S6 Fig). In the other case, isolates spanning the PBT are associated with a low expression cluster (E2) and with segments C1.1 and E1.6 (present in peptide 13). The E2 cluster is also however associated with segments C1.3 and E1.3 (present in peptide 14) whose isolates are all above the PBT. Peptides 13 and 14 differ by 11 amino acids with eight of these differences being in segment C. However, peptide 13 only differs from the vaccine antigen (peptide 1) in four of these positions with three in segment C, which suggests that the variation in segment C in combination with low expression could be responsible for the low RP values for isolates containing fHbp peptide 13.

### Assessment of sequence-based *fHbp* expression levels in vaccinated individuals

A key potential use for the *fHbp* expression cluster is to predict vaccine coverage for strains that have caused disease in individuals subject to immunization with an fHbp-containing vaccine and where case confirmation was by PCR with no viable culture. All PCR-only confirmed IMD cases routinely have their *fHbp* gene sequenced as part of the Public Health England enhanced surveillance of 4C-MenB [26]. The fHbp PCR product spans both the CDS and IGR and the routine sequencing of the *fHbp* CDS results in sequence data for the IGR (Borrow *et al*., personal communication). We were able, therefore, to perform a sequence-based analyses of *fHbp* IGR for all vaccine eligible cases (n = 49) of MenB disease occurring between introduction of 4C-MenB in September 2015 and January 2017 (Table 1). There were 20 cases in non-vaccinated individuals and 29 in individuals that had received one or two doses of 4C-MenB (these individuals are referred to as partially-vaccinated individuals as a three-dose regime is the licensed schedule for infants). The proportion of strains with variant 1 fHbp alleles was higher in non-vaccinees but not significantly different to the proportion in the partially-vaccinated individuals (71%, 12/17 and 56%, 15/27 for each group, respectively). In partially-vaccinated individuals, 53% (8/15) of typed strains had *fHbp* IGRs associated with the lowest two expression clusters (i.e. E1 and E2). Four of the other isolates had high expression IGR alleles (E5) but possessed low homology peptide 15 fHbp alleles. All MATS analyses of isolates from partially-vaccinated individuals harbouring variant 1 peptides (n = 8) produced RP values above the PBT of which four were due to fHbp IGR/peptide combinations that are associated with lack of coverage for fHbp as determined by our sequence-based comparison to MATS (Fig 6), two cases were due to strains that are always above the PBT and two with IGRs of an unknown expression level. Among the seven cases who had received either one (n = 4) or two (n = 3) doses of 4C-MenB and where confirmation was by PCR alone of a strain containing a variant 1 fHbp peptide, five of the invasive strains possessed fHbp peptide/IGR combinations that can be MATS negative and hence associated with lack of coverage.

**Table 1:** fHbp expression cluster and peptide type for MenB isolates from 4C-MenB vaccine breakthrough cases.

Number of 4C-MenB doses/cases	Diagnosis Method(cases)	fHbp variant/peptide/IGR/IGR Cluster/(cases)		
		MATS Covered^a^	MATS Not Covered^b^	Not Applicable
None/20 cases	Culture (3) or Culture and PCR (6)	1/13/3/E2 (3), 1/14/1/E2 (1)	1/13/3/E2 (1), 1/47/1/E2 (1), 2/19/1/E2 (1), 3/31/9/ND (1), 3/47/1/E2 (1)	
	PCR (11)			1/1/6/E4 (1), 1/13/3/E2 (2), 1/14/1 or 7/E2 (2), 1/322/1/E2 (1), 2/24/1/E2 (1), 3/499/9/ND (1), no data (3)
One dose/17 cases	Culture and PCR (7), culture (3)	1/13/3/E2 (2), 1/14/1/E2 (1)	2/19/i1/E2 (2), 3/various/various (5)	
	PCR (7)			1/15/4/E5 (3), 1/4/2/E1 (1), 2/19 or 202/1/E2 (2), no data (1)
At least 2/12 cases	Culture and PCR (6), culture (3)	1/4/2/E1 (1), 1/13/3/E2 (2), 1/954/24/ND (1), 1/510/28/ND (1)	3/45/33/ND (1), 3/953/1/E2 (1), 3/45/8/ND (1), No data (1)	
	PCR (3)			1/13/3/E2 (1), 1/15/4/E5 (1), 1/215/5/E3 (1)

^a^MATS Covered, relative potency greater than PBT for fHbp

^b^MATS Not Covered, relative potency equal to or less than PBT for fHbp

## Discussion

We have combined detection of multi-isolate IGR variation with quantitative and statistical tests of gene expression to understand the population variation in *fHbp* expression, a key meningococcal vaccine antigen. Analysis of natural variation in IGRs is readily detectable through multi-isolate whole genome sequence comparisons and is highly informative for understanding promoter function. We describe an *fHbp* expression cluster scheme that associates key polymorphic sites with variation in *fHbp* expression at the transcriptional level and utilise this scheme to develop a sequence-based approach to assessing strain coverage of a vaccine containing fHbp. Finally, this sequence-based approach was applied to analysis of cases of disease that have occurred in vaccine eligible infants since introduction of 4C-MenB into the UK national infant immunisation programme in September 2015.

We identified 110 IGRs for the *fHbp* gene and analysed expression for nine IGRs present in ~92% of England, Wales and Northern Ireland disease-causing strains carrying the variant 1 fHbp peptide contained in 4C-MenB. Hence our scheme covers a high proportion of meningococcal UK disease cases. Importantly, this scheme covers IGRs present in ST-269 sub-cluster isolates that are over-represented in disease cases where confirmation is based solely on PCR [27]. We performed comparisons to Western blot analyses with a polyclonal antisera specific for variant 1 fHbp. The polyclonal antisera produced a pattern that did not correlate with the qRT-PCR data but did correlate with peptide homology to the immunising antigen (S4 Fig). The lack of a correlation is likely due to the variable presence of antibody-reactive epitopes, and antibodies with a range of avidities for these epitopes, masking differences in protein expression levels. Our qRT-PCR overcomes this limitation by utilizing conserved binding sites for the primers so that the assay is not influenced by strain-to-strain variability in the target gene. The differential bias between the two techniques was clearly indicated for IGRs 4 and 10, which are classified as high expressers by qRT-PCR but low expressers by Western blotting with both IGRs being compromised by an association with peptide 15, which has only 85% homology to the immunizing antigen. In order to determine if our qRT-PCR measurements of *fHbp* expression were correlated with the levels of fHbp protein expression, we also compared our results to those of Biagini *et al*. [12] who quantified expression of the fHbp protein in whole cell lysates by mass spectrometry and associated these levels with *fHbp* IGR sequence. A similar pattern of expression was observed for qRT-PCR versus mass spectrometry (S7 Fig), indicating that our qRT PCR assay provides reliable measurements of relative protein expression for meningococcal isolates over a range of variant 1 fHbp peptide alleles and is robust for correlating IGR clusters with fHbp expression in rich media and standard growth conditions. Intriguingly, one exceptional isolate exhibited transcripts but an undetectable amount of protein. This fHbp peptide allele was only detected in four other clinical isolates (PubMLST *Neisseria* database April 2017). This rare allele is notable as it contains a single amino acid substitution relative to a highly expressed peptide, suggesting that post-translational regulation may significantly reduce protein expression in a small sub-set of fHbp peptides.

The statistical testing of the contributions of specific allelic variants to differences in gene expression identified specific sites that are likely to control gene expression. This statistical approach narrows the range of sites for targeting by a site-directed mutagenesis study of gene expression control. The statistical approach is however limited by the number of tested isolates such that significance for other sites may have been detected with a much larger amount of qRT-PCR data. Nevertheless, key variant sites were identified in IGR alleles 4, 6 and 10 that exhibit high fHbp expression and read-through transcription from *cbbA* into *fHbp*. These variant nucleotides were predicted to disrupt formation of a stem loop structure as detected by ARNold (data not shown) and noted by previous researchers as a potential terminator of read-through transcription for this locus [8]. The stem-loop structure is formed of two DNA uptake sequences that are found in other parts of the meningococcal genomes and hence the perturbation of expression observed herein may be relevant to expression of other meningococcal genes. Sanders *et al*. [10] reported that *cbbA* is induced in iron-restricted conditions in cc32 and other isolates and, although this gene was not detected as part of the *Nm* Fur regulon [28], these results suggest that fHbp expression will be significantly up-regulated by low iron conditions, as are likely to be prevalent both on mucosal surfaces and in blood, for strains that produce bicistronic *cbbA-fHbp* transcripts. As these *fHbp* IGR alleles are frequently present in hypervirulent cc32 and cc269 strains, this mode of regulation may contribute to their pathogenicity by elevating resistance to serum bactericidal killing. The other key regulatory allelic variant was present in a putative -35 sequence, this is indicative of the importance of the core promoter in determining gene expression levels or perhaps identifies variation that could modulate transcriptional regulation by Fnr, which binds to the adjacent 22 nucleotides according to DNA footprinting [8]. A similar analysis of the *cbbA* promoter also indicated that variation in the putative core promoter and Fur binding sites influenced transcript levels of this gene. A key insight from our study is that single nucleotide changes may have significant effects on expression of meningococcal proteins and hence phenotypic variation or immune-escape by lowered surface expression may be readily-accessible by both mutation and lateral gene transfer.

4C-MenB was predicted to protect against up to 88% of MenB strains with low fHbp expression being one determinant for a lack of coverage of certain strains. These coverage estimates were derived from MATS, a sandwich ELISA, and SBA assays. A drawback of these assays is the requirement for a viable meningococcal isolate whereas ~50% of IMD cases are confirmed by PCR only, without a viable culture. Since introduction of 4C-MenB into the UK national infant immunization programme, there have been a small number of cases of disease in partially-vaccinated vaccine eligible infants (i.e. those receiving one or two 4C-MenB doses) by MenB strains that contain vaccine-compatible variant 1 fHbp alleles. Currently, there is no method for assessing strain coverage of many of these cases and it is unclear whether IMD has occurred due to low immunity, lack of coverage as a result of low fHbp expression or another immune-evasive mutational change in the disease-causing strain.

We have assessed the utility of a sequence-based scheme for assessing 4C-MenB coverage of variant 1 fHbp alleles by comparing the combination of *fHbp* expression cluster and fHbp vaccine antigen homology levels to MATS RP scores. Critically, we detected evidence of compensatory effects of homology and expression levels. Thus, peptide allele 1.4 is associated with expression cluster E1 whose low expression level appears to be compensated by high (96%) homology to the vaccine antigen resulting in all isolates containing this peptide/IGR combination being above the PBT (i.e. the level required for protection as defined by MATS). Conversely, many isolates with the low homology (86%) peptide allele 15 are also above the PBT due to the compensatory effects of high expression from a cluster E5-associated *fHbp* IGR. We also observed that the differing ranges of RP scores in MATS of peptides 13 and 14 was not due to differing levels of expression as these peptides are within the same expression cluster and hence is more likely due to amino acid variation in one region of the fHbp peptide. The key prediction from our sequence-based scheme is that seven combinations of peptide/expression cluster are always associated with isolates above the MATS PBT while six combinations are ‘at risk’ of falling below the fHbp PBT (i.e. one or more isolates with the same combination are below the PBT). These ‘at risk’ combinations are 13/E2, 86/E5, 309/E2, 456/E2, 123/E2, 15/E5 and 622/E5. Assuming there is no variation in MATS RP score and hence in the potential for an isolate to fall below the PBT, the proportion of MATS–ve isolates for 13/E2 and 15/E5 were 33% (22/66) and 9% (3/32), respectively. The probabilities of the RP scores of fHbp peptides 13 and 15 falling below the PBT were 18% (9–30%, CI 95%) and 3% (0.1–16%, CI 95%), respectively, as compared to 0% for peptides 4 (0–4%, CI 95%) and 14 (0–13%, CI 95%) assuming a uniform data distribution. Higher values were obtained if the data was assumed to have a normal distribution. These probabilities provide an indication of the likelihood of strains with the 13/E2 and 15/E5 combinations exhibiting a lack of coverage as detected by MATS and are a measure of the ‘at risk’ coverage values for the sequence-based analyses of peptide/IGR sequence comparisons.

This scheme was applied to cases occurring in vaccine eligible infants in the UK since introduction of 4C-MenB into the national infant immunization programme. There were 15 cases with variant 1 fHbp alleles in individuals that had received one or two doses of 4C-MenB. For eight of the cases, a viable strain was obtained and found to be MATS positive for fHbp. For these eight cases, the sequence-based scheme was more conservative designating four as due to ‘at risk’ fHbp peptide/IGR combinations, two as indeterminate (as the IGRs were of unknown expression levels) and two as containing combinations associated with protective MATS values. The sequence-based scheme was also applied to the seven cases where confirmation was by PCR alone. Five of these cases were due to ‘at risk’ strains, one was indeterminate due to the presence of a rare peptide (215, 93% homologous to the vaccine antigen) of unknown expression level and one contained an 4/E1 fHbp peptide/IGR combination, although this latter case occurred in an individual receiving only one vaccine dose. This latter data set demonstrates the applicability of the *fHbp* IGR analysis to understanding vaccine strain coverage where confirmation is solely by PCR. Finally, this sequence-based scheme has the potential to replace the fHbp MATS assay due to the high portability, rapid turn-around and minimal cost.

In summary, we examined the association between IGR sequence and transcriptional expression for *fHbp* and defined clusters of IGR sequences with differing levels of expression. We have applied this scheme to interpretation of MATS predictions of fHbp coverage by 4C-MenB, a MenB vaccine, resulting in a sequence-based approach for predicting coverage of disease-causing strains by this vaccine antigen.

## Supporting information

S1 Fig Alignment of the 106 fHbp_IGR sequences. (DOCX)

S2 Fig Northern blot analysis of fhbp and cbba transcript levels. (PDF)

S3 Fig Alignment of 26 cbba_IGR sequences. (PDF)

S4 Fig Western blot analysis and relative quantity of fHbp. (PDF)

S5 Fig Combinatorial effects of expression level and homology to the Bexsero vaccine antigen for MATS-based estimates of fHbp strain coverage. This Fig depicts variations in MATS data and expression clusters for different segment C and E combinations of the fHbp protein.
(PDF)

S6 Fig Multalin alignment of the 12 fHbp peptide sequences present in the bacteria population tested. (PDF)

S7 Fig Comparison of RQ values obtained by qRT PCR and mass spectrometry for different fHbp IGR sequences. (PDF)

S1 Table Details of the 2008 *Neisseria meningitidis* genomes analysed. This file contains a spreadsheet with genome ID numbers, isolate information, alleles for the fHbp and cbba nucleotide and amino acid sequences, and homologies of the amino acid sequences to vaccine antigens.
(XLSX)

S2 Table Isolates tested for fHbp expression. (PDF)

S3 Table Primers and probes used in this study. (PDF)

S4 Table Nucleotide position in IGR alignment of fHbp. This file contains a spreadsheet that identifies the position of each polymorphic nucleotide in the *fHbp* IGR and the variable region code for these polymorphisms.
(XLSX)

S5 Table Regression model analysis of the contributions of variable region groups to expression of fHbp. (PDF)

S6 Table P-values from pairwise t-tests for comparison of mean fhbp RQ values of the fHbp IGR clusters detected by linear regression. (DOCX)

S7 Table P-values from pairwise t-tests for comparison of mean fhbp RQ values of fHbp expression clusters. (PDF)

S8 Table Comparison of fHbp MATS relative potency values, peptide and IGR expression cluster for 273 isolates. (PDF)

S1 Data Set MIQE information for the qRT-PCR assays. This file contains all of the optimization and validation data for the *fHbp* and *cbbA* qRT-PCR assays.
(PDF)

S2 Data Set Mathematical modelling of the agglomerative clustering and linear regression analyses of *fHbp* expression data. (PDF)

S3 Data Set Agglomerative clustering analysis of the cbbA qRT-PCR data and a probability analysis of the fHbp MATS threshold data for selected fHbp peptide/IGR combinations. (PDF)
