# Supplementary material for: Clustered intergenic region sequences as predictors of factor H Binding Protein expression patterns and for assessing Neisseria meningitidis strain coverage by meningococcal vaccines
Source: PLoS One. 2018 May 30;13(5):e0197186. doi: 10.1371/journal.pone.0197186 (PMC5976157; doi:10.1371/journal.pone.0197186)
Supplement: S1 Fig — (DOCX) [file pone.0197186.s001.docx]

**10 20 30 40 50 60 70 80 90 100 110 120 130 140 150 160 170 180 190 200**

**....|....|....|....|....|....|....|....|....|....|....|....|....|....|....|....|....|....|....|....|....|....|....|....|....|....|....|....|....|....|....|....|....|....|....|....|....|....|....|....|**

**1**  **CAGGTTGCTTGTAAACAAAATGCCGTCTGAACCGTTGTTCGGACGGCATTTGATTTTTGCTTCTTTGACCTGCCTCATTGATGCAGTATGC-AAAAAAAGATACCATAACCAAAATGTTTA-T-----------------------------------------------------------------------------**

**10**  **........C.........................CC......G..T.............................................--............GC........C.....-.-----------------------------------------------------------------------------**

**100** **........C.....................................T............................T........G......-............A................-.-----------------------------------------------------------------------------**

**101** **.........................................................................A...A.............-.............................-.-----------------------------------------------------------------------------**

**102** **...........................................................................................-...........................C.-.ATATTGAGACCTTTGCAATAACATAGGTTACTAAAATTTTATGCTCAATCTCATTTTCAAAATGCAAAACTTTTCTG**

**103** **........C.........................CCA...............................................G....A.-.....................G......T-.-----------------------------------------------------------------------------**

**104** **..................................................................................A........-.............................-.-----------------------------------------------------------------------------**

**105** **...........................................................................................-........................T....-.-----------------------------------------------------------------------------**

**106** **.......AC................C........CC.......................................................-.............GC........C.....-.-----------------------------------------------------------------------------**

**107** **..................................CC....................................T..................-............A................-.-----------------------------------------------------------------------------**

**108** **......................................................................................C....-.............................-.-----------------------------------------------------------------------------**

**109** **...........................................................................................-.............................-.-----------------------------------------------------------------------------**

**11**  **.....................A................................................................C....-.............GC........C.....-.-----------------------------------------------------------------------------**

**110** **........C.....................................T......................................A.....A.............GC........C.....-.-----------------------------------------------------------------------------**

**12**  **........CG........................CC.......................................................-.............GC........C.....-.-----------------------------------------------------------------------------**

**13**  **........CG..................A.....CC.......................................................-.............GC........C.....-.-----------------------------------------------------------------------------**

**14**  **.............................................A........................................C....-.............GC........C.....-.-----------------------------------------------------------------------------**

**15**  **..................................CC.......................................................-.............................-.-----------------------------------------------------------------------------**

**16**  **.......AC..................................................................................-.............................-.-----------------------------------------------------------------------------**

**17**  **..................................CC.......................................................-.............................-.-----------------------------------------------------------------------------**

**18**  **.................................................................................C.........-.............................-.-----------------------------------------------------------------------------**

**2**  **...........................................................................................-.............................-.ATATTGAGACCTTTGCAATAACATAGGTTACTAAAATTTTATGCTCAATCTCATTTTCAAAATGCAAAACTTTTCTG**

**20**  **........C.....................................T......................................A.....-.............GC........C.....-.-----------------------------------------------------------------------------**

**21**  **........C.....................................T............................T........G......-.............................-.-----------------------------------------------------------------------------**

**22**  **....................................................................................G....A.-.....................G......T-.-----------------------------------------------------------------------------**

**23**  **...........................................................................................-.............................-.-----------------------------------------------------------------------------**

**24**  **....................................................................T......................-.............................-.-----------------------------------------------------------------------------**

**25**  **........C.........................CC.......................................T........G......-....G........................-.-----------------------------------------------------------------------------**

**26**  **........C.........................CC................................................G....A.-.....................G......T-.-----------------------------------------------------------------------------**

**27**  **........C.......................T.............T......................................A.....-.............GC........C.....-.-----------------------------------------------------------------------------**

**28**  **........C.........................CC......G.................A.T............T...............-.............................-.-----------------------------------------------------------------------------**

**29**  **........C.........................CC.......................................................-.............................-.-----------------------------------------------------------------------------**

**3**  **...........................................................................................-.............................-.-----------------------------------------------------------------------------**

**30**  **...................................................A.......................................-.............................-.-----------------------------------------------------------------------------**

**31**  **........CG........................C........................---.............................-.............................-.-----------------------------------------------------------------------------**

**32**  **........C.........................CC.......................................T........G......-.............................-.-----------------------------------------------------------------------------**

**33**  **............................................A.........................................C....-.............GC........C.....-.-----------------------------------------------------------------------------**

**34**  **..................................CC.......................................................-.............................T.-----------------------------------------------------------------------------**

**35**  **.......AC.........................CC.......................................................-.....................G......T-.-----------------------------------------------------------------------------**

**36**  **.......AC.........................C.....................................T...........G....A.-.............................-.-----------------------------------------------------------------------------**

**37**  **...........................................................................................-.............................-.ATATTGAGACCTTTGCAATAACATAGGTTACTAAAATTTTATGCTCAATCTCATTTTCAAAATGCAAAACTTTTCTG**

**38**  **........CG........................CC..........T............................................-.............................-.-----------------------------------------------------------------------------**

**39**  **........CG.............T..........CC.......................................................-.............GC........C.....-.-----------------------------------------------------------------------------**

**4**  **........C.........................CC......G..T.............................................-.............GC........C.....-.-----------------------------------------------------------------------------**

**40**  **...........................................................................................-.............................-.-----------------------------------------------------------------------------**

**41**  **...........................................................................................-.............GC..............-.-----------------------------------------------------------------------------**

**42**  **......................T...............................................................C....-.............GC........C.....-.-----------------------------------------------------------------------------**

**43**  **........C.....................................T............................................-.............GC........C.....-.-----------------------------------------------------------------------------**

**44**  **.......AC..............T..........C.....................................T...........G....A.-.............................-.-----------------------------------------------------------------------------**

**45**  **..................................CC.......................................................-.............................-.-----------------------------------------------------------------------------**

**46**  **........CG........................CC.....A.................................................-.............GC........C.....-.-----------------------------------------------------------------------------**

**47**  **............................A.........................................................C....-.............GC........C.....-.-----------------------------------------------------------------------------**

**48**  **........CG......................T.CC..........T......................................A.....-.............GC........C.....-.-----------------------------------------------------------------------------**

**49**  **..............................................T......................................A.....-.............GC........C.....-.-----------------------------------------------------------------------------**

**5**  **.......AC.........................CC................................................G....A.-.....................G......T-.-----------------------------------------------------------------------------**

**50**  **...........................................................................................-.............................-.ATATTGAGACCTTTGCAATAACATAGGTTACTAAAATTTTATGCTCAATCTCATTTTCAAAATGCAAAACTTTTCTG**

**51**  **...........................................................................................-.....................G......T-.-----------------------------------------------------------------------------**

**52**  **...........................................................................................-.............................-.-----------------------------------------------------------------------------**

**53**  **........CG........................CC.......................---......................G....A.-.....................G......T-.-----------------------------------------------------------------------------**

**54**  **.........................C............................................................C....-.............GC........C.....-.-----------------------------------------------------------------------------**

**55**  **...........................................................................................--............................-.-----------------------------------------------------------------------------**

**59**  **.......AC.....................................T......................................A.....-.............GC........C.....-.-----------------------------------------------------------------------------**

**6**  **........C.........................CC.........A......................................G......-.............................-.-----------------------------------------------------------------------------**

**60**  **.........................C........CC................................................G....A.--....................G......T-.-----------------------------------------------------------------------------**

**61**  **------------------------------------------------......................................C....-.............GC........C.....-.-----------------------------------------------------------------------------**

**62**  **.........................-------------................................................C....-.............GC........C.....-.-----------------------------------------------------------------------------**

**63**  **......A.C.........................C-................................................G......-.......T.............G.......-.-----------------------------------------------------------------------------**

**64**  **.......................T..........CC...........................-...........................-.............................-.-----------------------------------------------------------------------------**

**65**  **...........................................................................................-.............................-.-----------------------------------------------------------------------------**

**66**  **........C.........................CC................................T...T..................-.............................-.-----------------------------------------------------------------------------**

**67**  **............................................C.........................................C....-.............GC........C.....-.-----------------------------------------------------------------------------**

**68**  **..................................CC......G..T.............................................-.............GC........C.....-.-----------------------------------------------------------------------------**

**69**  **........C..............T......................T......................................A.....-.............GC........C.....-.-----------------------------------------------------------------------------**

**7**  **...........................................................................................-.............................-.-----------------------------------------------------------------------------**

**70**  **........CG................A.......CC.......................................................-.............GC........C.....-.-----------------------------------------------------------------------------**

**71**  **........CG......................T.CC..........T............................................-.............................-.-----------------------------------------------------------------------------**

**72**  **........C.........................CC.............................................C.........-.............................-.-----------------------------------------------------------------------------**

**73**  **..................................CC......G................................................-.............................-.-----------------------------------------------------------------------------**

**74**  **.......AC...G..A..................CC.......................................................-.............................T.-----------------------------------------------------------------------------**

**75**  **........C............A................................................................C....-.............GC........C.....-.-----------------------------------------------------------------------------**

**76**  **...........................................................................................-.............................-.-----------------------------------------------------------------------------**

**77**  **........C.........................CC.........A......................................G......-.............................-.-----------------------------------------------------------------------------**

**78**  **..........................G...........................................................C....-.............GC........C.....-.-----------------------------------------------------------------------------**

**79**  **..............................................G.......................................C....-.............GC........C.....-.-----------------------------------------------------------------------------**

**8**  **......................................................................................C....-.............GC........C.....-.-----------------------------------------------------------------------------**

**80**  **..................................CC.............................................C.........-.............................-.-----------------------------------------------------------------------------**

**81**  **........C...............A.........CC......G.................A.T............T...............-.............................-.-----------------------------------------------------------------------------**

**82**  **..................................CC.....................................T.......C.........-.............................-.-----------------------------------------------------------------------------**

**83**  **.......................G..............................................................C....-.............GC........C.....-.-----------------------------------------------------------------------------**

**84**  **........C.........................CCA......................................................-...................G.........-.-----------------------------------------------------------------------------**

**85**  **.................................................................................C.........-.............................-.-----------------------------------------------------------------------------**

**86**  **........C.......C.................CC......GT........G.........T............................A.................T...........-.-----------------------------------------------------------------------------**

**87**  **........CG........................CC.........A.............................................-.............GC........C.....-.-----------------------------------------------------------------------------**

**88**  **.....................A..................................................T.........A.G....A.-.............................-.-----------------------------------------------------------------------------**

**89**  **..................................................G...................................C....-.............GC........C.....-.-----------------------------------------------------------------------------**

**9**  **........C.....................................T......................................A.....-.............GC........C.....-.-----------------------------------------------------------------------------**

**90**  **........CG........................CC..........T............................................-.............GC........C.....-.-----------------------------------------------------------------------------**

**91**  **........................A.............................................................C....-.............GC........C.....-.-----------------------------------------------------------------------------**

**92**  **..................................CC....................................T..................-.............................-.-----------------------------------------------------------------------------**

**93**  **....C...C.........................CC......G.........................T...T..................A.............................-.-----------------------------------------------------------------------------**

**94**  **.......................T..............................................................C....-.............GC........C.....-.-----------------------------------------------------------------------------**

**95**  **........C.........................CC.......................................................-............A.....T.GGG......-.-----------------------------------------------------------------------------**

**96**  **...........................................................................................-.............................-.-----------------------------------------------------------------------------**

**97**  **........C..................................................................................-.............................-.-----------------------------------------------------------------------------**

**98**  **........C.......................T.CC.......................................................-.............................-.-----------------------------------------------------------------------------**

**99**  **........C.........................CCA...................................T...........G....A.-.............................-.-----------------------------------------------------------------------------**

**210 220 230 240 250 260 270 280 290 300 310 320 330 340 350 360**

**....|....|....|....|....|....|....|....|....|....|....|....|....|....|....|....|....|....|....|....|....|....|....|....|....|....|....|....|....|....|....|....|....|..**

**1**  **--------------------------------------------------------------------------------------------------------------ATATTATCTAT---------------------TCTGCGTATGACTAGGAGCAAACCT**

**10**  **--------------------------------------------------------------------------------------------------------------...........---------------------....T.............T......**

**100** **--------------------------------------------------------------------------------------------------------------...........---------------------..................T......**

**101** **--------------------------------------------------------------------------------------------------------------...........---------------------.........................**

**102** **ATTTTTCCTACTTTTTGCTCAATATTAGGAAGGTTTTTAGGCAATTGAAAATTTTTTGGCGCATTTTTATGCGTCAAATTTCGTTAACAGACTATTTTTGCAAAGGTCTC...........---------------------.........................**

**103** **--------------------------------------------------------------------------------------------------------------...........---------------------..................T......**

**104** **--------------------------------------------------------------------------------------------------------------...........---------------------.........................**

**105** **--------------------------------------------------------------------------------------------------------------...........---------------------.........................**

**106** **--------------------------------------------------------------------------------------------------------------...........---------------------....T.............T......**

**107** **--------------------------------------------------------------------------------------------------------------...........---------------------.........................**

**108** **--------------------------------------------------------------------------------------------------------------...........---------------------.........................**

**109** **----------------------------------------------------------------------------------------------------------------------------------------------------...................**

**11**  **--------------------------------------------------------------------------------------------------------------...........---------------------....T.............T......**

**110** **--------------------------------------------------------------------------------------------------------------...........---------------------....T.............T......**

**12**  **--------------------------------------------------------------------------------------------------------------...........---------------------....T.............T......**

**13**  **--------------------------------------------------------------------------------------------------------------...........---------------------....T.............T......**

**14**  **--------------------------------------------------------------------------------------------------------------...........---------------------....T.............T......**

**15**  **--------------------------------------------------------------------------------------------------------------...........---------------------.........................**

**16**  **--------------------------------------------------------------------------------------------------------------...........---------------------..................T......**

**17**  **--------------------------------------------------------------------------------------------------------------...........---------------------.....A...................**

**18**  **--------------------------------------------------------------------------------------------------------------...........---------------------.........................**

**2**  **ATTTTTCCTACTTTTTGCTCAATATTAGGAAGGTTTTTAGGCAATTGAAAATTTTTTGGCGCATTTTTATGCGTCAAATTTCGTTAACAGACTATTTTTGCAAAGGTCTC...........---------------------.........................**

**20**  **--------------------------------------------------------------------------------------------------------------...........TCTGTGTATGACTAGATCTAT..................T......**

**21**  **--------------------------------------------------------------------------------------------------------------...........---------------------..................T......**

**22**  **--------------------------------------------------------------------------------------------------------------...........---------------------..................T......**

**23**  **--------------------------------------------------------------------------------------------------------------...........---------------------.....A...................**

**24**  **--------------------------------------------------------------------------------------------------------------...........---------------------.........................**

**25**  **--------------------------------------------------------------------------------------------------------------...........---------------------..................T......**

**26**  **--------------------------------------------------------------------------------------------------------------...........---------------------..................T......**

**27**  **--------------------------------------------------------------------------------------------------------------...........---------------------....T.............T......**

**28**  **--------------------------------------------------------------------------------------------------------------...........---------------------..................T......**

**29**  **--------------------------------------------------------------------------------------------------------------...........---------------------.........................**

**3**  **--------------------------------------------------------------------------------------------------------------...........---------------------..................T......**

**30**  **--------------------------------------------------------------------------------------------------------------....C......---------------------.........................**

**31**  **--------------------------------------------------------------------------------------------------------------...........---------------------............A.....T......**

**32**  **--------------------------------------------------------------------------------------------------------------...........---------------------..................T......**

**33**  **--------------------------------------------------------------------------------------------------------------...........---------------------....T.............T......**

**34**  **--------------------------------------------------------------------------------------------------------------...........---------------------.........................**

**35**  **--------------------------------------------------------------------------------------------------------------...........---------------------....T.............T......**

**36**  **--------------------------------------------------------------------------------------------------------------...........---------------------..................T......**

**37**  **ATTTTTCCTACTTTTTGCTCAATATTAGGAAGG-TTTTAGGCAATTGAAAATTTTTTGGCGCATTTTTATGCGTCAAATTTCGTTAACAGACTATTTTTGCAAAGGTCTC...........---------------------.........................**

**38**  **--------------------------------------------------------------------------------------------------------------...........---------------------.........................**

**39**  **--------------------------------------------------------------------------------------------------------------...........---------------------....T.............T......**

**4**  **--------------------------------------------------------------------------------------------------------------...........---------------------....T.............T......**

**40**  **-----------------------------------------------------------------------------------------------------------ATT...........---------------------.........................**

**41**  **--------------------------------------------------------------------------------------------------------------...........---------------------.........................**

**42**  **--------------------------------------------------------------------------------------------------------------...........---------------------....T.............T......**

**43**  **--------------------------------------------------------------------------------------------------------------...........---------------------....T.............T......**

**44**  **--------------------------------------------------------------------------------------------------------------...........---------------------..................T......**

**45**  **--------------------------------------------------------------------------------------------------------------...........---------------------..................T......**

**46**  **--------------------------------------------------------------------------------------------------------------...........---------------------....T.............T......**

**47**  **--------------------------------------------------------------------------------------------------------------...........---------------------....T.............T......**

**48**  **--------------------------------------------------------------------------------------------------------------...........---------------------....T.............T......**

**49**  **--------------------------------------------------------------------------------------------------------------...........---------------------....T.............T......**

**5**  **--------------------------------------------------------------------------------------------------------------...........---------------------..................T......**

**50**  **ATTTTTCCTACTTTTTGCTCAATATTAGGAAGGTTTTTAGGCAATTGAAAATTTTTTGGCGCATTTTTATGCGTCAAATTTCGTTAACAGACTATTTTTGCAAAGGTCTC...........---------------------....T....................**

**51**  **--------------------------------------------------------------------------------------------------------------...........---------------------..................T......**

**52**  **----------------------------------------------------------------------------------------------------------------.........---------------------.........................**

**53**  **--------------------------------------------------------------------------------------------------------------...........---------------------..................T......**

**54**  **--------------------------------------------------------------------------------------------------------------...........---------------------....T.............T......**

**55**  **--------------------------------------------------------------------------------------------------------------...........---------------------.........................**

**59**  **--------------------------------------------------------------------------------------------------------------...........---------------------....T.............T......**

**6**  **--------------------------------------------------------------------------------------------------------------...........---------------------..................T......**

**60**  **--------------------------------------------------------------------------------------------------------------...........---------------------..................T......**

**61**  **--------------------------------------------------------------------------------------------------------------...........---------------------....T.............T......**

**62**  **--------------------------------------------------------------------------------------------------------------...........---------------------....T.............T......**

**63**  **--------------------------------------------------------------------------------------------------------------.-------------------------------.....A............T......**

**64**  **--------------------------------------------------------------------------------------------------------------...........---------------------...A.....................**

**65**  **--------------------------------------------------------------------------------------------------------------.....G.....---------------------.........................**

**66**  **--------------------------------------------------------------------------------------------------------------...........---------------------.........................**

**67**  **--------------------------------------------------------------------------------------------------------------...........---------------------....T.............T......**

**68**  **--------------------------------------------------------------------------------------------------------------...........---------------------....T.............T......**

**69**  **--------------------------------------------------------------------------------------------------------------...........---------------------....T.............T......**

**7**  **--------------------------------------------------------------------------------------------------------------....C......---------------------.........................**

**70**  **--------------------------------------------------------------------------------------------------------------...........---------------------....T.............T......**

**71**  **--------------------------------------------------------------------------------------------------------------...........---------------------.........................**

**72**  **--------------------------------------------------------------------------------------------------------------...........---------------------.........................**

**73**  **--------------------------------------------------------------------------------------------------------------...........---------------------.........................**

**74**  **--------------------------------------------------------------------------------------------------------------...........---------------------.T................T......**

**75**  **--------------------------------------------------------------------------------------------------------------...........---------------------....T.............T......**

**76**  **--------------------------------------------------------------------------------------------------------------....C......---------------------..................T......**

**77**  **--------------------------------------------------------------------------------------------------------------..G........---------------------..................T......**

**78**  **--------------------------------------------------------------------------------------------------------------...........---------------------....T.............T......**

**79**  **--------------------------------------------------------------------------------------------------------------...........---------------------....T.............T......**

**8**  **--------------------------------------------------------------------------------------------------------------...........---------------------....T.............T......**

**80**  **--------------------------------------------------------------------------------------------------------------...........---------------------.........................**

**81**  **--------------------------------------------------------------------------------------------------------------...........---------------------..................T......**

**82**  **--------------------------------------------------------------------------------------------------------------...........---------------------.........................**

**83**  **--------------------------------------------------------------------------------------------------------------...........---------------------....T.............T......**

**84**  **--------------------------------------------------------------------------------------------------------------...........---------------------..................T......**

**85**  **--------------------------------------------------------------------------------------------------------------...........---------------------..................T......**

**86**  **--------------------------------------------------------------------------------------------------------------...........---------------------..................T......**

**87**  **--------------------------------------------------------------------------------------------------------------...........---------------------....T.............T......**

**88**  **--------------------------------------------------------------------------------------------------------------...........---------------------..................T......**

**89**  **--------------------------------------------------------------------------------------------------------------...........---------------------....T.............T......**

**9**  **--------------------------------------------------------------------------------------------------------------...........---------------------....T.............T......**

**90**  **--------------------------------------------------------------------------------------------------------------...........---------------------....T.............T......**

**91**  **--------------------------------------------------------------------------------------------------------------...........---------------------....T.............T......**

**92**  **--------------------------------------------------------------------------------------------------------------...........---------------------...A.....................**

**93**  **--------------------------------------------------------------------------------------------------------------...........---------------------...A..............T......**

**94**  **--------------------------------------------------------------------------------------------------------------...........---------------------....T.............T......**

**95**  **--------------------------------------------------------------------------------------------------------------...........--------------------A..................T......**

**96**  **--------------------------------------------------------------------------------------------------------------...........---------------------...A..............T......**

**97**  **-------------------------------------------------------------------------------------------------------------T...........---------------------.........................**

**98**  **--------------------------------------------------------------------------------------------------------------...........---------------------..................T......**

**99**  **--------------------------------------------------------------------------------------------------------------...........---------------------..................T......**

Supplementary Figure 1. Alignment of the 106 fHbp_IGR sequences. The dots indicate that the sequence is similar at this position to the first sequence of the alignment. Hyphens represent a gap in the alignment.
