# Supplementary material for: Clustered intergenic region sequences as predictors of factor H Binding Protein expression patterns and for assessing Neisseria meningitidis strain coverage by meningococcal vaccines
Source: PLoS One. 2018 May 30;13(5):e0197186. doi: 10.1371/journal.pone.0197186 (PMC5976157; doi:10.1371/journal.pone.0197186)
Supplement: S2 Fig — (PDF) [file pone.0197186.s002.pdf]

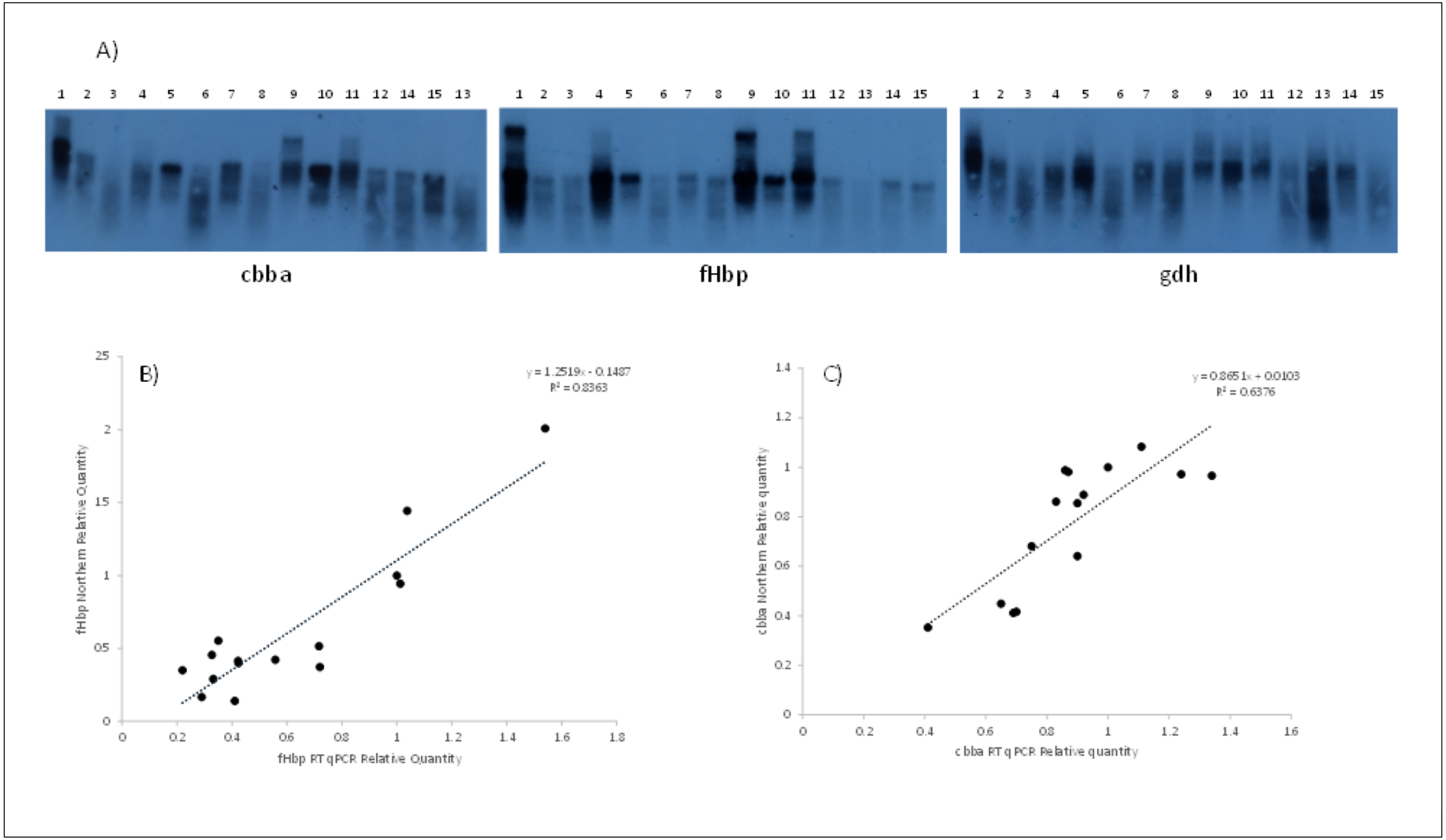

Supplementary Figure 2. Northern blot analysis of *fhbp* and *cbba* transcript levels. A) Northern blot results for *cbba*, *fhbp* and *gdh* transcripts. From 1 to 15 the clinical isolates are:- 1, H44/76 (cc32); 2, M11 241044 (cc41/44); 3, M11 240290 (cc213); 4, M12 240203 (cc162); 5, M13 240048 (cc269); 6, M13

240134 (cc198); 7, M12 240679 (cc11); 8, M12 240237 (cc23); 9, M13 240245 (cc269); 10, M13 240189 (cc11); 11, M13 240486 (cc269); 12, M14 240018 (cc41/44); 13, M04 240731 (cc41/44); 14, M15 240147 (cc41/44); 15, M11 240247 (cc11).. B) *fhbp* RQs measured for each isolate by northern analysis were compared to the RQs measured by RT qPCR. The dashed line represents the best fit line. C) *cbba* RQs measured for each isolate by northern analysis were compared to the RQs measured by RT qPCR. The dashed line represents the line of best fit.
