# Supplementary material for: Clustered intergenic region sequences as predictors of factor H Binding Protein expression patterns and for assessing Neisseria meningitidis strain coverage by meningococcal vaccines
Source: PLoS One. 2018 May 30;13(5):e0197186. doi: 10.1371/journal.pone.0197186 (PMC5976157; doi:10.1371/journal.pone.0197186)
Supplement: S3 Fig — (PDF) [file pone.0197186.s003.pdf]

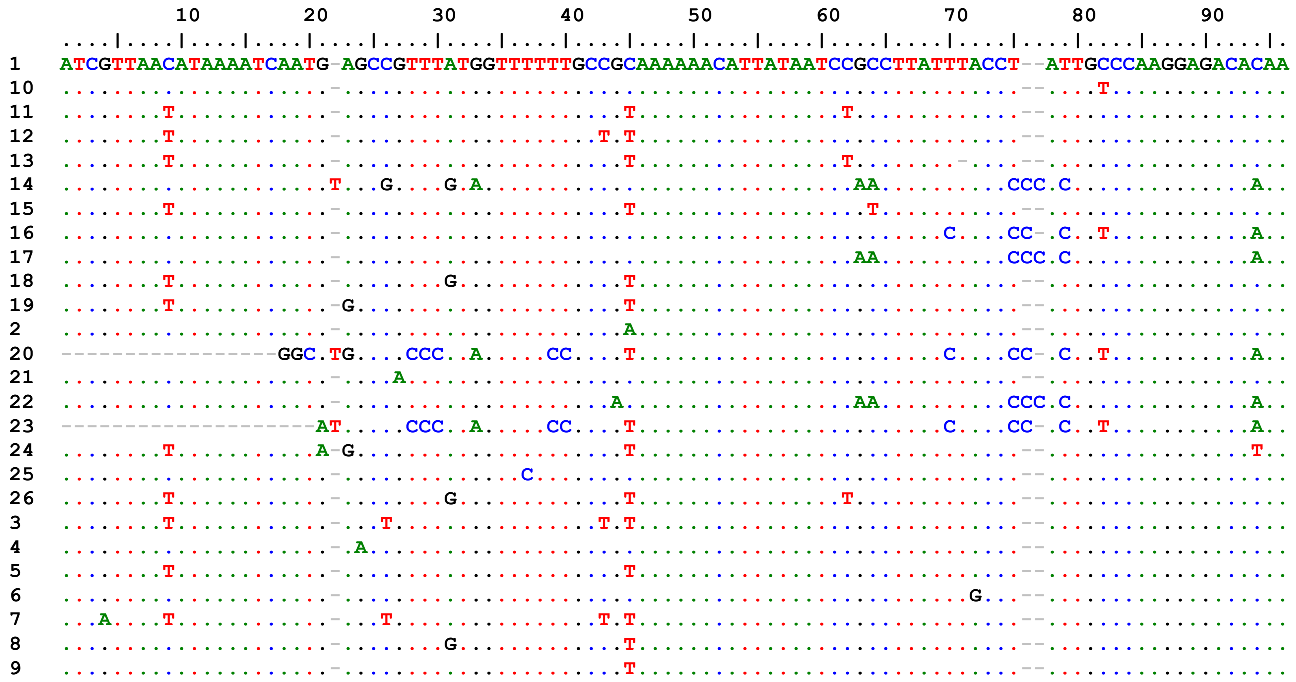

Supplementary Figure 3. Alignment of 26 cbba_IGR sequences. The dots indicate that the sequence is similar at this position to the first sequence of the alignment. Hyphens represent a gap in the alignment.
