# Supplementary material for: Clustered intergenic region sequences as predictors of factor H Binding Protein expression patterns and for assessing Neisseria meningitidis strain coverage by meningococcal vaccines
Source: PLoS One. 2018 May 30;13(5):e0197186. doi: 10.1371/journal.pone.0197186 (PMC5976157; doi:10.1371/journal.pone.0197186)
Supplement: S4 Fig — (PDF) [file pone.0197186.s004.pdf]

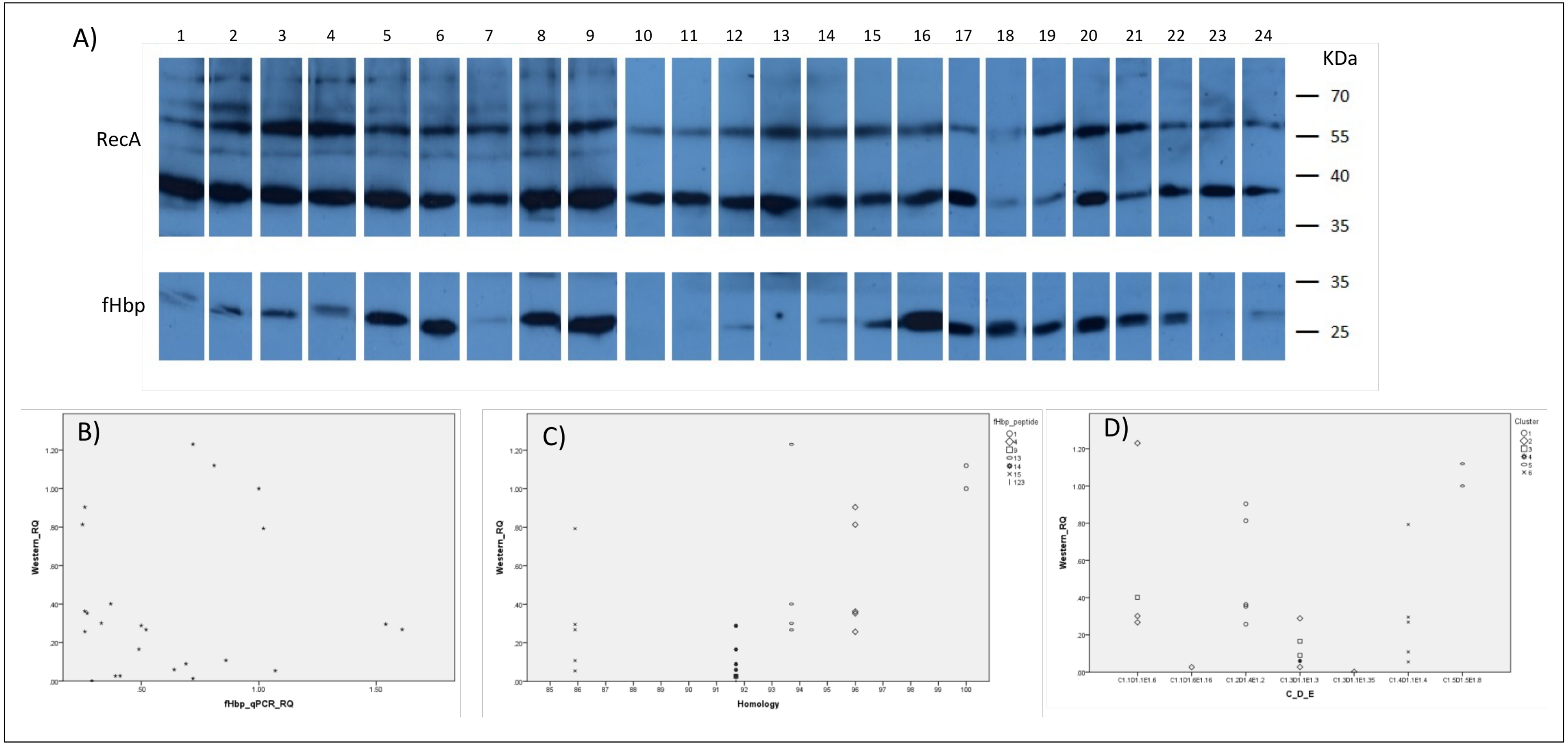

Supplementary Figure 4. Western blot analysis and relative quantity of fHbp. A) Western blot analysis of fHbp and RecA. B) Comparison of the relative quantities(RQ) of fHbp measured by RT qPCR and Western blot. The samples tested are (lane 1 to 24): M14 240472, M14 240434, 3-M13 240245, M13 240336, M11 241066, M11 240175, M11 240409, M11 240149, H44/76, M11 240247, M15 240147, M02 240210, M04 240731, M14 240476, M14 240477, M14 240367, M04 241215, M13 240189, M12 240846, M13 240048, M12 240202, M11 240236, M11 240389, M11 240766. Western blot fHbp RQ values stratified by percentage of homology with fHbp peptide 1 (C) or the combination of fHbp segment subtypes (D).
