# Supplementary material for: Clustered intergenic region sequences as predictors of factor H Binding Protein expression patterns and for assessing Neisseria meningitidis strain coverage by meningococcal vaccines
Source: PLoS One. 2018 May 30;13(5):e0197186. doi: 10.1371/journal.pone.0197186 (PMC5976157; doi:10.1371/journal.pone.0197186)
Supplement: S5 Fig — This Fig depicts variations in MATS data and expression clusters for different segment C and E combinations of the fHbp protein. (PDF) [file pone.0197186.s005.pdf]

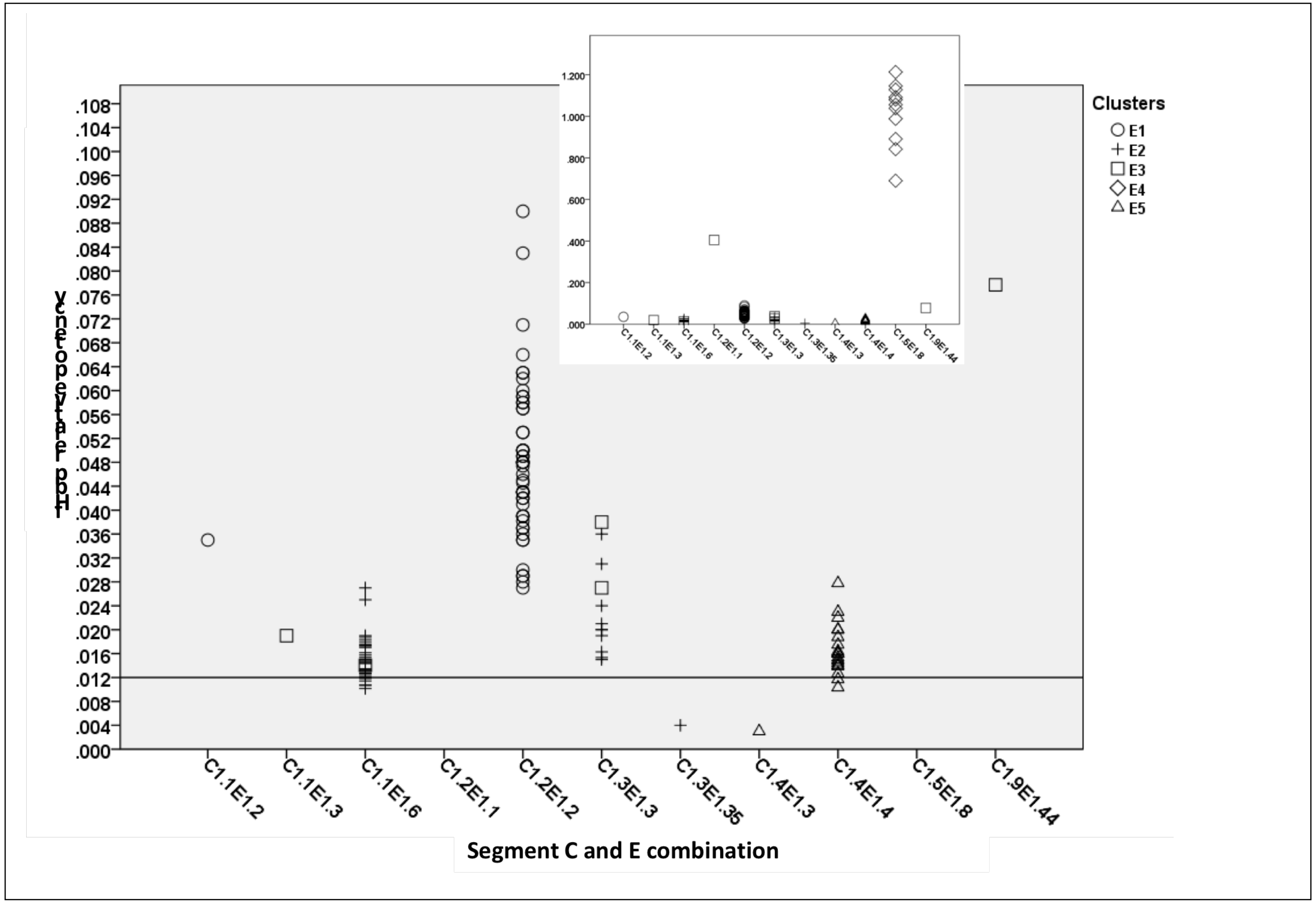

Supplementary Figure 5. Combinatorial effects of expression level and homology to the Bexsero vaccine antigen for MATS-based estimates of fHbp strain coverage. All isolates (n = 132) were MenB strains with variant 1 fHbp alleles. Each symbol represents one isolate. The y-axis is the relative potency (RP) score obtained in an fHbp MATS assay. The dash lined represents the fHbp PBT below which an isolate is defined as negative for coverage by fHbp. The x-axis contains the combination of C and E segments present in the fHbp peptide sequence as defined by Beernick *et al.* (2009)[18]. Expression clusters (E1 to E5) are represented by specific symbols as indicated.
