# Supplementary material for: Clustered intergenic region sequences as predictors of factor H Binding Protein expression patterns and for assessing Neisseria meningitidis strain coverage by meningococcal vaccines
Source: PLoS One. 2018 May 30;13(5):e0197186. doi: 10.1371/journal.pone.0197186 (PMC5976157; doi:10.1371/journal.pone.0197186)
Supplement: S6 Fig — (PDF) [file pone.0197186.s006.pdf]

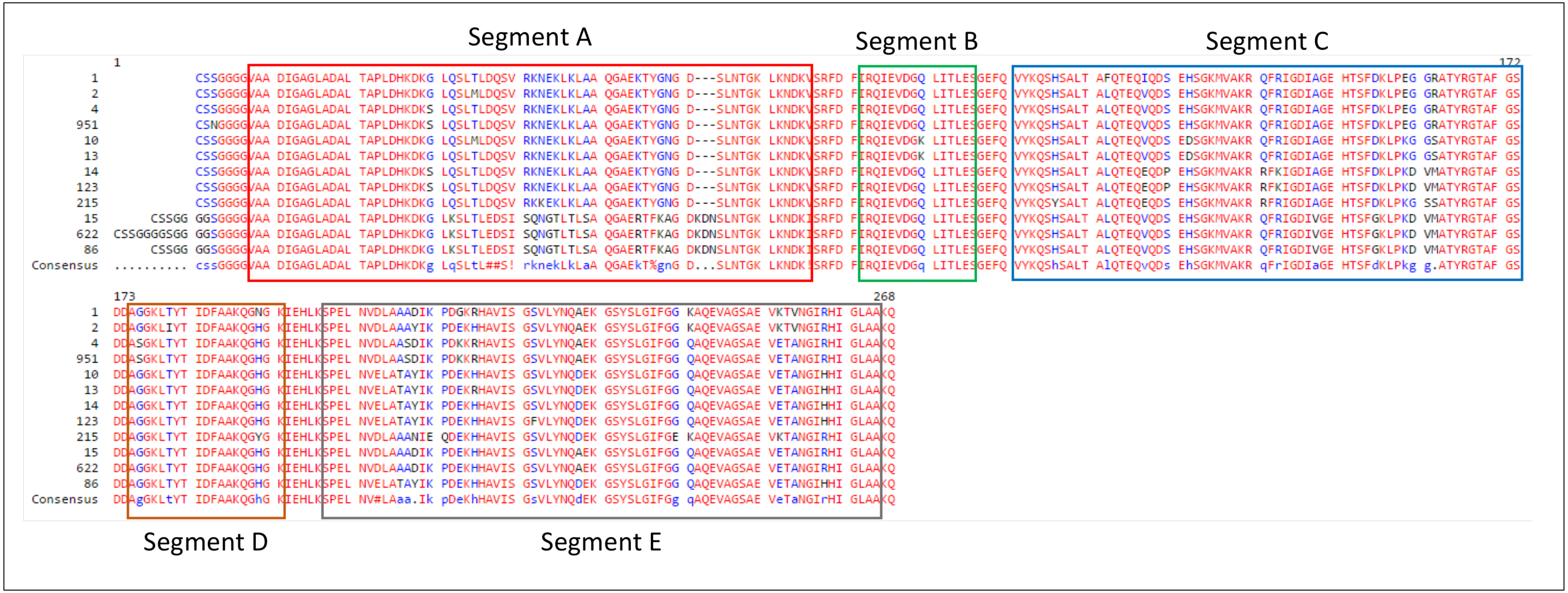

Supplementary figure 6. Multalin alignment of the 12 fHbp peptide sequences present in the bacteria population tested. The squares indicate the position of the segment as described by Beernink et al. 2009 and Pajon et al. 2010.
