# Supplementary material for: Clustered intergenic region sequences as predictors of factor H Binding Protein expression patterns and for assessing Neisseria meningitidis strain coverage by meningococcal vaccines
Source: PLoS One. 2018 May 30;13(5):e0197186. doi: 10.1371/journal.pone.0197186 (PMC5976157; doi:10.1371/journal.pone.0197186)
Supplement: S7 Fig — (PDF) [file pone.0197186.s007.pdf]

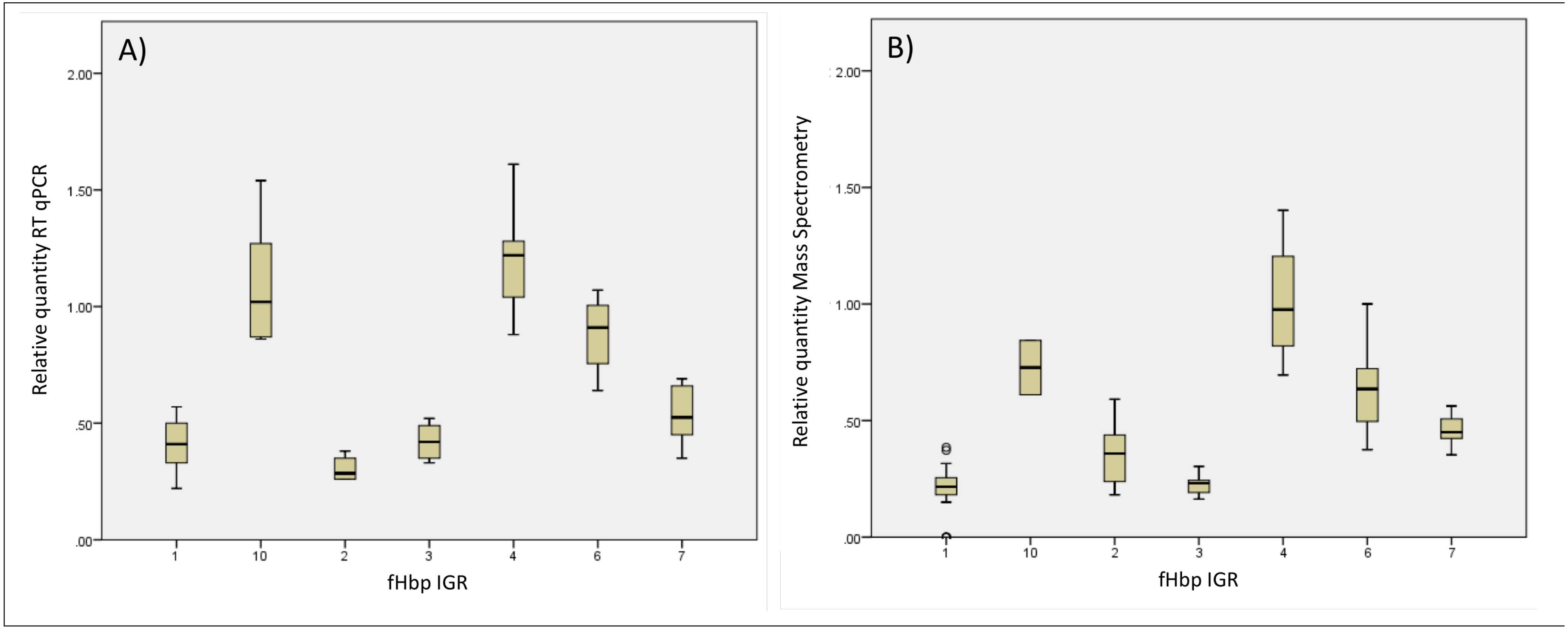


**Supplementary Figure 7.** Comparison of RQ values obtained by qRT PCR and mass spectrometry for different fHbp IGR sequences. A) qRT-PCR RQ value (n = 52). B) Mass spectrometry RQ values (n = 71) derived from Biagini *et al* 2012. The Nm isolates studied are different between the two analyses.
