# Supplementary material for: Clustered intergenic region sequences as predictors of factor H Binding Protein expression patterns and for assessing Neisseria meningitidis strain coverage by meningococcal vaccines
Source: PLoS One. 2018 May 30;13(5):e0197186. doi: 10.1371/journal.pone.0197186 (PMC5976157; doi:10.1371/journal.pone.0197186)
Supplement: S2 Table — (PDF) [file pone.0197186.s009.pdf]

Supplementary Table 2. Isolates tested for fHbp expression.

BIGSdb ID	Isolate	Clonal complex	Sequence type	Serogroup	fHbp peptide	Cbba peptide	fHbp forward Primer	CBBA_IGR_	FHBP_IGR_	fHbp RQ Vs H44/76	cbba RQ vs H44/76	Bicistronic transcript*	IGR cluster
38142	M04 240731	cc41/44	ST-340	B	123	1	229T	1	1	0·29	0·87	0	E2
19993	M10 240569	cc18	ST-18	B	37	1	229T	1	1	0·44	0·9	0	E2
19997	M10 240579	NA	ST-3687	B	4	38	229T	1	1	0·33	1·55	0	E2
20166	M11 240002	NA	ST-4954	B	14	1	229T	1	1	0·41	0·91	0	E2
20285	M11 240165	cc174	ST-14	Y	254	1	229T	1	1	0·27	1·18	0	E2
20424	M11 240389	cc11	ST-11	W	9	1	229T	1	1	0·39	0·99	0	E2
21160	M11 240723	NA	ST-1345	B	14	46	229T	1	1	0·36	0·8	0	E2
21211	M11 240946	cc269	ST-1161	B	4	1	229T	1	1	0·55	1·35	0	E2
27778	M12 240336	cc41/44	ST-1475	B	14	1	229T	1	1	0·5	0·91	0	E2
28109	M13 240134	cc198	ST-823	NG	4	1	229T	1	1	0·22	0·86	0	E2
35463	M13 240614	cc41/44	ST-10868	B	4	10	229T	1	1	0·57	1·4	0	E2
35492	M13 240675	cc41/44	ST-1475	B	14	1	229T	1	1	0·53	0·77	0	E2
35741	M14 240368	NA	ND	B	14	1	229T	1	1	0·45	0·63	0	E2
37906	M15 240147	cc41/44	ST-9352	B	14	1	229T	1	1	0·41	0·69	0	E2
38143	M04 241215	cc41/44	ST-41	B	4	1	229T	1	2	0·27	0·99	0	E1
20277	M11 240149	cc41/44	ST-2314	B	4	1	229T	1	2	0·26	1·21	0	E1
21257	M11 241044	cc41/44	ST-1194	B	4	1	229T	1	2	0·35	0·9	0	E1
27833	M12 240741	cc41/44	ST-8054	B	4	6	229T	1	2	0·38	1·26	0	E1
34517	M13 240525	cc41/44	ST-41	B	4	2	229T	1	2	0·3	0·62	0	E1
35795	M14 240477	cc41/44	ST-485	B	4	1	229T	1	2	0·26	0·53	0	E1
20322	M11 240236	cc269	ST-1161	B	13	3	229T	1	3	0·52	0·89	0	E2
21289	M12 240006	cc269	ST-1161	B	13	3	229T	1	3	0·35	0·73	0	E2
27930	M13 240048	cc269	ST-1161	B	13	3	229T	1	3	0·33	0·65	0	E2
34506	M13 240490	cc269	ST-5972	B	13	3	229T	1	3	0·42	0·85	0	E2
35783	M14 240465	NA	ST-11306	B	13	3	229T	1	3	0·49	0·87	0	E2
38130	M01 240007	cc269	ST-269	B	15	4	229T	2	4	1·32	1·35	1	E5

38131	M01 240601	cc269	ST-1791	B	15	2	229T	1	4	1·28	1·23	1	E5
38137	M02 240210	cc269	ST-1195	B	15	4	229T	2	4	1·07	1·03	1	E5
38140	M03 240823	cc269	ST-1284	B	15	4	229T	2	4	0·88	0·86	1	E5
20235	M11 240077	cc269	ST-269	B	15	4	229T	2	4	0·98	0·97	1	E5
21248	M11 241033	cc269	ST-269	B	15	4	229T	2	4	1·22	1·14	1	E5
27867	M12 240831	cc269	ST-1049	B	15	4	229T	2	4	1·28	1·19	1	E5
34504	M13 240486	cc269	ST-269	B	15	4	229T	2	4	1·04	1·11	1	E5
35774	M14 240434	cc269	ST-269	B	15	4	229T	2	4	1·61	1·28	1	E5
30049	M02 241729	cc11	ST-11	B	812	7	229T	1	5	0·61	1·34	0	E3
20066	M10 240684	cc11	ST-11	B	601	16	229T	1	5	0·45	1·05	0	E3
20075	M10 240701	cc213	ST-213	B	89	27	229C	5	5	0·68	0·95	0	E3
20249	M11 240108	cc41/44	ST-2632	B	14	9	229T	1	5	0·56	0·87	0	E3
20329	M11 240247	cc11	ST-11	C	-	7	229T	1	5	0·72	1·24	0	E3
21232	M11 240994	cc11	ST-11	C	650	7	229T	1	5	0·63	1·1	0	E3
21253	M11 241039	cc11	ST-11	C	13	7	229T	1	5	0·55	1·26	0	E3
21474	M12 240290	cc41/44	ST-340	B	14	9	229T	1	5	0·42	0·7	0	E3
28083	M12 240698	cc11	ST-11	C	13	7	229T	1	5	0·69	0·85	0	E3
27947	M13 240090	cc41/44	ST-6761	B	14	9	229T	1	5	0·52	0·91	0	E3
28097	M13 240254	cc11	ST-11	C	13	7	229T	1	5	0·71	1·53	0	E3
28103	M13 240402	cc11	ST-11	C	13	7	229T	1	5	0·48	0·85	0	E3
35542	M14 240008	cc41/44	ST-11265	B	14	7	229T	1	5	0·7	0·84	0	E3
30213	M14 240094	NA	ND	C	13	7	229T	1	5	0·76	0·98	0	E3
35749	M14 240383	cc32	ST-33	B	10	61	229T	1	5	0·67	1·03	0	E3
35794	M14 240476	cc41/44	ST-340	B	14	9	229T	1	5	0·64	1·18	0	E3
30171	M98 252111	cc11	ST-11	C	131	7	229T	1	5	0·62	1·37	0	E3
20477	H44/76	cc32	ST-32	B	1	5	229C	3	6	1	1	1	E4
20292	M11 240175	cc32	ST-33	B	1	5	229C	3	6	0·81	0·83	1	E4
21420	M12 240203	cc162	ST-1	B	1	5	229C	3	6	1·01	0·75	1	E4
27912	M13 240017	cc32	ST-10707	B	1	5	229C	3	6	0·64	0·48	1	E4
35546	M14 240014	cc32	ST-32	B	1	5	229C	7	6	1·07	0·78	1	E4

35797	M14 240480	cc32	ST-11302	B	1	5	229C	3	6	0·91	0·82	1	E4
240	MC58	cc32	ST-74	B	1	5	229C	3	6	0·7	0·61	1	E4
38135	M01 241601	cc41/44	ST-42	B	14	1	229T	1	7	0·35	0·47	0	E2
20109	M10 240750	cc41/44	ST-8203	B	14	1	229T	1	7	0·66	1·48	0	E2
21184	M11 240766	cc41/44	ST-8054	B	14	1	229T	1	7	0·69	1·28	0	E2
27791	M12 240656	cc41/44	ST-3695	B	14	1	229T	1	7	0·45	0·58	0	E2
35550	M14 240018	cc41/44	ST-6782	B	14	1	229T	1	7	0·56	0·92	0	E2
35790	M14 240472	cc41/44	ST-8054	B	14	1	229T	1	7	0·49	0·82	0	E2
20439	M11 240409	cc269	ST-269	B	15	4	229T	2	10	0·86	1·12	1	E5
21418	M12 240201	cc269	ST-269	B	15	4	229T	2	10	1·27	1·57	1	E5
27937	M13 240072	cc269	ST-269	B	15	4	229T	2	10	0·87	0·85	1	E5
28016	M13 240245	cc269	ST-269	B	15	4	229T	2	10	1·54	1·34	1	E5
35740	M14 240367	cc269	ST-269	B	15	4	229T	2	10	1·02	1·01	1	E5
21256	M11 241043	cc11	ST-11	C	13	6	229T	1	16	0·45	0·63	0	E2
21419	M12 240202	cc11	ST-11	C	13	6	229T	1	16	0·37	0·64	0	E2
28082	M12 240679	cc11	ST-11	C	13	6	229T	1	16	0·42	0·9	0	E2
28095	M13 240189	cc11	ST-11	C	13	6	229T	1	16	0·72	0·83	0	E2
26732	M13 240559	cc11	ST-11	C	13	6	229T	1	16	0·47	1·3	0	E2
21273	M11 241066	cc41/44	ST-41	B	4	1	229T	6	19	0·25	0·48	0	E1
27872	M12 240846	cc41/44	ST-41	B	4	1	229T	6	19	0·26	0·29	0	E1
27998	M13 240210	cc41/44	ST-41	B	4	1	229T	6	19	0·31	0·57	0	E1
28014	M13 240237	cc41/44	ST-41	B	4	1	229T	6	19	0·33	0·41	0	E1
28076	M13 240479	cc41/44	ST-41	B	4	1	229T	6	19	0·35	0·27	0	E1

*0=absent; 1=present.
