# Supplementary material for: Clustered intergenic region sequences as predictors of factor H Binding Protein expression patterns and for assessing Neisseria meningitidis strain coverage by meningococcal vaccines
Source: PLoS One. 2018 May 30;13(5):e0197186. doi: 10.1371/journal.pone.0197186 (PMC5976157; doi:10.1371/journal.pone.0197186)
Supplement: S3 Table — (PDF) [file pone.0197186.s010.pdf]

Supplementary Table 3. Primers and probes used in this study.

Primer Name	Target gene	Primer/probe sequence	Assay	origin
FHbp_229C_F	*fHbp*	ACTTTATCCGCCAAATCGAAGT	qPCR	This study
FHbp_229T_F	*fHbp*	ACTTTATCCGTCAAATCGAAGT	qPCR	This study
FHbp_313_R	*fHbp*	GCGGAATGGCTTTGTTTGTA	qPCR	This study
FHbp_253_P	*fHbp*	[6FAM]ACGGGIAGCTCATTACCTTGGAGA[BHQ1]	qPCR	This study
IGF	Intergenic region	ACAAAATGCCGTCTGAAC	qPCR	Sanders et al. 2012
IGR	Intergenic region	CATCAATGAGGCAGGTCA	qPCR	Sanders et al. 2012
16S_Fw	16S	ACGGAGGGTGCGAGCGTTAATC	qPCR	Echenique-Rivera 2011
16S_Rv	16S	CTGCCTTCGCCTTCGGTATTCCT	qPCR	Echenique-Rivera 2011
gdh350F	*gdh*	TCGCCATTAAAGCCGAAATC	qPCR	Sanders et al. 2012
gdh416R2	*gdh*	TTGCCGGTACGCAGGTAGA	qPCR	Modified from Sanders et al. 2012
gdh374T	*gdh*	[JOE]ACGAACGCTGGAAGGGCGTTC[BHQ1]	qPCR	Sanders et al. 2012
nmb1869F	*cbba*	GGAGACACAAATGGCACTCGTA	qPCR	Sanders et al. 2012
nmb1869R	*cbba*	GGCAGGCCGTAGCTGTTTT	qPCR	Sanders et al. 2012
nmb1869P	*cbba*	[6FAM]CATGCGCCAACTGCTTGATCATGC[BHQ1]	qPCR	Sanders et al. 2012
fHbp404_NP_F	*fHbp*	CGGGCGAACATACATCTTTT	PCR/Northern	This study
fHbp642_NP_R	*fHbp*	GTAAAGGACGAAACCGCTGA	PCR/Northern	This study
cbba637_NP_F	*cbba*	CGTATCGACCGCATCAAAGA	PCR/Northern	This study
cbba887_NP_R	*cbba*	GGGTTTTCGGCAAGGTAGC	PCR/Northern	This study
gdh123_NP_F	*gdh*	GCAAAACCACTTGATGCAAA	PCR/Northern	This study
gdh375_NP_R	*gdh*	TTCGATTTCGGCTTTAATGG	PCR/Northern	This study
