# Supplementary material for: Clustered intergenic region sequences as predictors of factor H Binding Protein expression patterns and for assessing Neisseria meningitidis strain coverage by meningococcal vaccines
Source: PLoS One. 2018 May 30;13(5):e0197186. doi: 10.1371/journal.pone.0197186 (PMC5976157; doi:10.1371/journal.pone.0197186)
Supplement: S5 Table — (PDF) [file pone.0197186.s012.pdf]

Supplementary Table 5. Regression model analysis of the contributions of variable region groups to expression of fHbp.

	Coefficients	*Standard Error*	*t Stat*	*P-value*	*Lower 95%*	*Upper 95%*	*Lower 95.0%*	*Upper 95.0%*
Intercept	1.160	0.038	30.356	0.000	1.084	1.236	1.084	1.236
V1[1]	-0.265	0.064	-4.124	0.000	-0.393	-0.137	-0.393	-0.137
V2[1]	-0.310	0.095	-3.258	0.002	-0.499	-0.120	-0.499	-0.120
V3[1]	-0.409	0.098	-4.161	0.000	-0.605	-0.213	-0.605	-0.213
V7[1]	-0.283	0.066	-4.274	0.000	-0.415	-0.151	-0.415	-0.151
V15[1]	-0.139	0.052	-2.694	0.009	-0.242	-0.036	-0.242	-0.036

[1]V, variable region groups; groups of polymorphisms in the fHbp IGR (see Supplementary Table 4)
