# Supplementary material for: Clustered intergenic region sequences as predictors of factor H Binding Protein expression patterns and for assessing Neisseria meningitidis strain coverage by meningococcal vaccines
Source: PLoS One. 2018 May 30;13(5):e0197186. doi: 10.1371/journal.pone.0197186 (PMC5976157; doi:10.1371/journal.pone.0197186)
Supplement: S6 Table — (DOCX) [file pone.0197186.s013.docx]

**Supplementary Table 6.** P-values from pairwise t-tests for comparison of mean *fhbp* RQ values of the fHbp IGR clusters detected by linear regression.

| **IGR Clusters^1^** | **ic20** | **ic16** | **ic21** | **ic5** | **ic6** | **ic22** |
| --- | --- | --- | --- | --- | --- | --- |
| **ic20** |  | 0.0371 | 0.0000 | 0.0000 | 0.0001 | 0.0000 |
| **ic16** | 0.0371 |  | 0.5213^2^ | 0.1082^2^ | 0.0013 | 0.0000 |
| **ic21** | 0.0000 | 0.5213^2^ |  | 0.0000 | 0.0002 | 0.0000 |
| **ic5** | 0.0000 | 0.1082^2^ | 0.0000 |  | 0.0043 | 0.0000 |
| **ic6** | 0.0001 | 0.0013 | 0.0002 | 0.0043 |  | 0.0057 |
| **ic22** | 0.0000 | 0.0000 | 0.0000 | 0.0000 | 0.0057 |  |

^1^IGR clusters consist of the following fHbp IGRs:- ic20, i2 and i19; ic21, i1, i3 and i7; ic22, i4 and i10; ic16, i16, ic5, i5; ic6, i6. ^2^Not statistically significant.
