# Supplementary material for: Clustered intergenic region sequences as predictors of factor H Binding Protein expression patterns and for assessing Neisseria meningitidis strain coverage by meningococcal vaccines
Source: PLoS One. 2018 May 30;13(5):e0197186. doi: 10.1371/journal.pone.0197186 (PMC5976157; doi:10.1371/journal.pone.0197186)
Supplement: S7 Table — (PDF) [file pone.0197186.s014.pdf]

Supplementary Table 7. P-values from pairwise t-tests for comparison of mean *fhbp* RQ values of fHbp expression clusters.

	**fHbp_IGR**	**E1**	**E2**	**E3**	**E4**	**E5**
**E1**	**2, 19**		0.0000	0.0000	0.0001	0.0000
**E2**	**1, 3, 7, 16**	0.0000		0.0000	0.0002	0.0000
**E3**	**5**	0.0000	0.0000		0.0043	0.0000
**E4**	**6**	0.0001	0.0002	0.0043		0.0057
**E5**	**4, 10**	0.0000	0.0000	0.0000	0.0057	
