# Supplementary material for: Clustered intergenic region sequences as predictors of factor H Binding Protein expression patterns and for assessing Neisseria meningitidis strain coverage by meningococcal vaccines
Source: PLoS One. 2018 May 30;13(5):e0197186. doi: 10.1371/journal.pone.0197186 (PMC5976157; doi:10.1371/journal.pone.0197186)
Supplement: S8 Table — (PDF) [file pone.0197186.s015.pdf]

Supplementary Table 8. Comparison of fHbp MATS relative potency values, peptide and IGR expression cluster for 273 isolates.

Isolate	fHbp Peptide (percentage of homology with peptide 1)	IGR	Cluster	fHbp relative potency
M14 240380	4 (96%)	2	1	0.040
M14 240388	4 (96%)	2	1	0.035
M14 240410	4 (96%)	2	1	0.047
M14 240425	4 (96%)	2	1	0.050
M14 240429	4 (96%)	2	1	0.061
M14 240455	4 (96%)	2	1	0.033
M14 240477	4 (96%)	2	1	0.028
M14 240516	4 (96%)	2	1	0.048
M14 240518	4 (96%)	2	1	0.057
M14 240533	4 (96%)	2	1	0.054
M14 240543	4 (96%)	2	1	0.043
M14 240551	4 (96%)	2	1	0.055
M14 240565	4 (96%)	2	1	0.047
M14 240595	4 (96%)	2	1	0.043
M14 240625	4 (96%)	2	1	0.053
M14 240631	4 (96%)	2	1	0.050
M14 240636	4 (96%)	2	1	0.045
M14 240639	4 (96%)	2	1	0.052
M15 240034	4 (96%)	2	1	0.043
M15 240067	4 (96%)	2	1	0.077
M15 240075	4 (96%)	2	1	0.060
M15 240087	4 (96%)	2	1	0.037
M15 240106	4 (96%)	2	1	0.042
M15 240155	4 (96%)	2	1	0.060
M15 240159	4 (96%)	2	1	0.044
M15 240176	4 (96%)	2	1	0.043
M15 240209	4 (96%)	2	1	0.044
M15 240225	4 (96%)	2	1	0.038
M15 240232	4 (96%)	2	1	0.040
M15 240262	4 (96%)	2	1	0.060
M15 240322	4 (96%)	2	1	0.033
M15 240333	4 (96%)	2	1	0.048
M15 240337	4 (96%)	2	1	0.048
M15 240357	4 (96%)	2	1	0.051
M15 240439	4 (96%)	2	1	0.042
M15 240445	4 (96%)	2	1	0.042
M15 240497	4 (96%)	2	1	0.050
M15 240499	4 (96%)	2	1	0.059
M15 240514	4 (96%)	2	1	0.044
M15 240552	4 (96%)	2	1	0.060
M15 240573	4 (96%)	2	1	0.037
M15 240737	4 (96%)	2	1	0.043

M15 240743	4 (96%)	2	1	0.083
M15 240745	4 (96%)	2	1	0.062
M15 240748	4 (96%)	2	1	0.043
M15 240775	4 (96%)	2	1	0.066
M15 240784	4 (96%)	2	1	0.048
M15 240799	4 (96%)	2	1	0.063
M15 240849	4 (96%)	2	1	0.090
M15 240877	4 (96%)	2	1	0.039
M15 240883	4 (96%)	2	1	0.030
M15 240902	4 (96%)	2	1	0.058
M15 240923	4 (96%)	2	1	0.059
M15 240939	4 (96%)	2	1	0.035
M15 240969	4 (96%)	2	1	0.038
M15 240973	4 (96%)	2	1	0.058
M15 240979	4 (96%)	2	1	0.039
M15 240986	4 (96%)	2	1	0.063
M15 240990	4 (96%)	2	1	0.029
M15 240996	4 (96%)	2	1	0.053
M16 240007	4 (96%)	2	1	0.053
M16 240046	4 (96%)	2	1	0.042
M16 240049	4 (96%)	2	1	0.039
M16 240059	4 (96%)	2	1	0.050
M16 240068	4 (96%)	2	1	0.060
M16 240155	4 (96%)	2	1	0.048
M16 240156	4 (96%)	2	1	0.048
M16 240160	4 (96%)	2	1	0.045
M16 240162	4 (96%)	2	1	0.057
M16 240187	4 (96%)	2	1	0.042
M16 240188	4 (96%)	2	1	0.043
M16 240209	4 (96%)	2	1	0.037
M16 240210	4 (96%)	2	1	0.045
M16 240229	4 (96%)	2	1	0.071
M16 240230	4 (96%)	2	1	0.048
M16 240257	4 (96%)	2	1	0.041
M16 240275	4 (96%)	2	1	0.029
M16 240276	4 (96%)	2	1	0.027
M16 240288	4 (96%)	2	1	0.043
M16 240289	4 (96%)	2	1	0.046
M16 240398	4 (96%)	2	1	0.049
M16 240414	4 (96%)	2	1	0.050
M16 240423	4 (96%)	2	1	0.037
M16 240432	4 (96%)	2	1	0.036
M16 240433	4 (96%)	2	1	0.035
M16 240434	4 (96%)	2	1	0.057
M16 240507	4 (96%)	2	1	0.050
M16 240532	4 (96%)	2	1	0.047

M16 240534	4 (96%)	2	1	0.049
M04 241215	4 (96%)	2	1	0.059
M14 240400	608 (95.6%)	2	1	0.056
M14 240643	889 (95.6%)	2	1	0.033
M16 240277	951 (95.6%)	2	1	0.035
M04 240731	123 (91.3%)	1	2	0.004
M15 240302	125 (93.3%)	1	2	0.026
M14 240390	13 (93.7%)	3	2	0.010
M14 240428	13 (93.7%)	3	2	0.015
M14 240437	13 (93.7%)	3	2	0.020
M14 240456	13 (93.7%)	3	2	0.007
M14 240465	13 (93.7%)	3	2	0.012
M14 240561	13 (93.7%)	3	2	0.021
M14 240563	13 (93.7%)	3	2	0.018
M14 240566	13 (93.7%)	3	2	0.014
M14 240641	13 (93.7%)	3	2	0.012
M14 240646	13 (93.7%)	3	2	0.012
M14 240647	13 (93.7%)	3	2	0.012
M15 240002	13 (93.7%)	3	2	0.013
M15 240014	13 (93.7%)	3	2	0.017
M15 240058	13 (93.7%)	3	2	0.016
M15 240060	13 (93.7%)	3	2	0.018
M15 240069	13 (93.7%)	3	2	0.029
M15 240071	13 (93.7%)	3	2	0.010
M15 240084	13 (93.7%)	3	2	0.012
M15 240090	13 (93.7%)	3	2	0.010
M15 240133	13 (93.7%)	3	2	0.017
M15 240175	13 (93.7%)	3	2	0.026
M15 240208	13 (93.7%)	3	2	0.017
M15 240235	13 (93.7%)	3	2	0.018
M15 240240	13 (93.7%)	3	2	0.020
M15 240253	13 (93.7%)	3	2	0.015
M15 240270	13 (93.7%)	3	2	0.018
M15 240281	13 (93.7%)	3	2	0.013
M15 240286	13 (93.7%)	3	2	0.012
M15 240325	13 (93.7%)	3	2	0.009
M15 240373	13 (93.7%)	3	2	0.012
M15 240437	13 (93.7%)	3	2	0.011
M15 240443	13 (93.7%)	3	2	0.011
M15 240468	13 (93.7%)	3	2	0.007
M15 240476	13 (93.7%)	3	2	0.012
M15 240541	13 (93.7%)	3	2	0.014
M15 240783	13 (93.7%)	3	2	0.011
M15 240789	13 (93.7%)	3	2	0.015
M15 240854	13 (93.7%)	3	2	0.019
M15 240859	13 (93.7%)	3	2	0.011

M15 240886	13 (93.7%)	3	2	0.010
M15 240897	13 (93.7%)	3	2	0.015
M15 240901	13 (93.7%)	3	2	0.013
M15 240912	13 (93.7%)	3	2	0.012
M15 240961	13 (93.7%)	3	2	0.011
M15 240975	13 (93.7%)	3	2	0.013
M15 240984	13 (93.7%)	3	2	0.013
M16 240019	13 (93.7%)	3	2	0.013
M16 240045	13 (93.7%)	3	2	0.014
M16 240058	13 (93.7%)	3	2	0.018
M16 240091	13 (93.7%)	3	2	0.016
M16 240121	13 (93.7%)	3	2	0.013
M16 240145	13 (93.7%)	3	2	0.013
M16 240159	13 (93.7%)	3	2	0.018
M16 240169	13 (93.7%)	3	2	0.016
M16 240199	13 (93.7%)	3	2	0.025
M16 240212	13 (93.7%)	3	2	0.015
M16 240244	13 (93.7%)	3	2	0.012
M16 240292	13 (93.7%)	3	2	0.012
M16 240390	13 (93.7%)	3	2	0.018
M16 240410	13 (93.7%)	3	2	0.017
M16 240426	13 (93.7%)	3	2	0.017
M16 240443	13 (93.7%)	3	2	0.019
M16 240519	13 (93.7%)	3	2	0.017
M16 240520	13 (93.7%)	3	2	0.014
M16 240549	13 (93.7%)	3	2	0.027
M02 240039	13 (93.7%)	3	2	0.015
M14 240387	14 (91.7%)	1	2	0.020
M14 240407	14 (91.7%)	7	2	0.019
M14 240471	14 (91.7%)	7	2	0.043
M14 240472	14 (91.7%)	7	2	0.036
M14 240541	14 (91.7%)	1	2	0.023
M14 240564	14 (91.7%)	7	2	0.028
M15 240063	14 (91.7%)	7	2	0.022
M15 240147	14 (91.7%)	1	2	0.015
M15 240147	14 (91.7%)	1	2	0.015
M15 240183	14 (91.7%)	1	2	0.015
M15 240296	14 (91.7%)	1	2	0.028
M15 240356	14 (91.7%)	7	2	0.022
M15 240474	14 (91.7%)	7	2	0.021
M15 240925	14 (91.7%)	1	2	0.015
M16 240056	14 (91.7%)	1	2	0.021
M16 240146	14 (91.7%)	1	2	0.020
M16 240178	14 (91.7%)	1	2	0.020
M16 240452	14 (91.7%)	1	2	0.031
M16 240512	14 (91.7%)	7	2	0.024

M16 240530	14 (91.7%)	1	2	0.016
M01 241601	14 (91.7%)	7	2	0.019
M14 240514	309 (92.1%)	1	2	0.012
M15 240297	327 (91.3%)	1	2	0.017
M14 240637	37 (93.7%)	1	2	0.054
M15 240307	37 (93.7%)	1	2	0.049
M15 240139	456 (92.1%)	1	2	0.012
M15 240180	700 (91.3%)	7	2	0.026
M14 240383	10 (92.9%)	5	3	0.019
M16 240494	13 (93.7%)	5	3	0.014
M14 240476	14 (91.7%)	5	3	0.038
M14 240490	14 (91.7%)	5	3	0.025
M15 240171	14 (91.7%)	5	3	0.035
M15 240289	14 (91.7%)	5	3	0.030
M15 240328	14 (91.7%)	5	3	0.038
M16 240211	14 (91.7%)	5	3	0.027
M15 240259	2 (96.8%)	5	3	0.337
M15 240744	2 (96.8%)	5	3	0.405
M15 240168	215 (93.3%)	5	3	0.073
M16 240036	215 (93.3%)	5	3	0.078
M14 240520	61 (94.9%)	5	3	0.108
M14 240539	61 (94.9%)	5	3	0.075
M15 240037	890 (93.3%)	5	3	0.022
M14 240467	1 (100%)	6	4	0.858
M14 240480	1 (100%)	6	4	0.543
M14 240527	1 (100%)	6	4	1.017
M14 240528	1 (100%)	6	4	0.785
M14 240560	1 (100%)	6	4	0.931
M14 240576	1 (100%)	6	4	0.612
M14 240591	1 (100%)	6	4	0.830
M14 240606	1 (100%)	6	4	0.891
M14 240623	1 (100%)	6	4	0.986
M15 240001	1 (100%)	6	4	0.993
M15 240020	1 (100%)	6	4	0.394
M15 240043	1 (100%)	6	4	1.034
M15 240113	1 (100%)	6	4	0.840
M15 240142	1 (100%)	6	4	1.005
M15 240233	1 (100%)	6	4	1.066
M15 240279	1 (100%)	6	4	1.017
M15 240304	1 (100%)	6	4	1.078
M15 240574	1 (100%)	6	4	0.860
M15 240747	1 (100%)	6	4	1.146
M15 240781	1 (100%)	6	4	0.891
M15 240802	1 (100%)	6	4	1.128
M15 240855	1 (100%)	6	4	0.690
M16 240042	1 (100%)	6	4	1.092

M16 240044	1 (100%)	6	4	1.056
M16 240218	1 (100%)	6	4	0.988
M16 240225	1 (100%)	6	4	0.842
M16 240243	1 (100%)	6	4	1.080
M16 240418	1 (100%)	6	4	1.039
M16 240566	1 (100%)	6	4	1.213
M14 240408	15 (85.9%)	4	5	0.015
M14 240434	15 (85.9%)	4	5	0.016
M14 240452	15 (85.9%)	4	5	0.014
M14 240466	15 (85.9%)	4	5	0.011
M14 240531	15 (85.9%)	10	5	0.020
M14 240542	15 (85.9%)	4	5	0.024
M14 240617	15 (85.9%)	4	5	0.016
M14 240626	15 (85.9%)	4	5	0.017
M15 240088	15 (85.9%)	4	5	0.022
M15 240102	15 (85.9%)	4	5	0.014
M15 240109	15 (85.9%)	10	5	0.016
M15 240122	15 (85.9%)	10	5	0.015
M15 240179	15 (85.9%)	10	5	0.013
M15 240182	15 (85.9%)	4	5	0.015
M15 240196	15 (85.9%)	4	5	0.012
M15 240241	15 (85.9%)	4	5	0.017
M15 240385	15 (85.9%)	4	5	0.015
M15 240424	15 (85.9%)	4	5	0.012
M15 240570	15 (85.9%)	10	5	0.014
M15 240760	15 (85.9%)	4	5	0.018
M15 240773	15 (85.9%)	10	5	0.012
M15 240835	15 (85.9%)	4	5	0.016
M15 240869	15 (85.9%)	4	5	0.028
M15 240934	15 (85.9%)	4	5	0.014
M16 240033	15 (85.9%)	4	5	0.015
M16 240071	15 (85.9%)	4	5	0.022
M16 240076	15 (85.9%)	4	5	0.014
M16 240113	15 (85.9%)	4	5	0.019
M16 240251	15 (85.9%)	4	5	0.013
M16 240396	15 (85.9%)	4	5	0.016
M16 240438	15 (85.9%)	4	5	0.015
M16 240496	15 (85.9%)	10	5	0.014
M01 240007	15 (85.9%)	4	5	0.020
M01 240601	15 (85.9%)	4	5	0.015
M02 240210	15 (85.9%)	4	5	0.023
M03 240823	15 (85.9%)	4	5	0.016
M06 241112	15 (85.9%)	4	5	0.020
M15 240466	622 (84.3%)	4	5	0.009
M16 240061	622 (84.3%)	4	5	0.010
M14 240469	825 (85.9%)	4	5	0.023

M16 240087	86 (84%)	4	5	0.003
