# Supplementary material for: Clustered intergenic region sequences as predictors of factor H Binding Protein expression patterns and for assessing Neisseria meningitidis strain coverage by meningococcal vaccines
Source: PLoS One. 2018 May 30;13(5):e0197186. doi: 10.1371/journal.pone.0197186 (PMC5976157; doi:10.1371/journal.pone.0197186)
Supplement: S1 Data Set — This file contains all of the optimization and validation data for the fHbp and cbbA qRT-PCR assays. (PDF) [file pone.0197186.s016.pdf]

OPTIMISATION OF FHBP GENE EXPRESSION ANALYSIS.

qPCR assay validation

Primer selection and in silico analysis

The primers and probe amplifying *cbbA* and the reference gene *gdh* were previously designed and published by Sanders et al. (2012). The primers were nonetheless checked for their universality among the bacterial population. For this purpose the primer sequences were compared to all *gdh* and *cbbA* alleles in the pubMLST sequence database. It was observed that in some cases the *gdh* reverse primer exhibited a mismatch in its first position. It was then decided to modify the reverse primer by removing this first nucleotide.

The *fHbp* gene primers and probes were designed using Primer 3 online software based on the alignment of all variant 1 *fHbp* alleles. For *fHbp* the only acceptable set included a mismatch in the middle of the forward primer. So two forward primers were designed for fHbp (Table 1).

Table 1. qPCR primer information.

Target gene	Primer Name	direction	sequence	Concentration in assay	Product size (bp)	origin
gdh	gdh350F	Forward	TCGCCATTAAAGCCGAAATC	200nM	68	Sanders et al. 2012
	gdh417R2	Reverse	TTGCCGGTACGCAGGTAGA	200nM		
	gdh374T	Probes	[JOE]ACGAACGCTGGAAGGGCGTTC[BHQ1]	150nM		
fHbp	FHbp_229_FC	Forward	ACTTTATCCGCCAAATCGAAGT	400nM	84	This Study
	FHbp_229_FT	Forward	ACTTTATCCGTCAAATCGAAGT	400nM		
	FHbp_313_R	Reverse	GCGGAATGGCTTTGTTTGTA	400nM		
	FHbp_253_P	Probe	[6FAM]ACGGGIAGCTCATTACCTTGGAGA[BHQ1]	200nM		
cbbA	nmb1869F	Forward	GGAGACACAAATGGCACTCGTA	300nM	72	Sanders et al. 2012
	nmb1869R	Reverse	GGCAGGCCGTAGCTGTTTT	300nM		
	nmb1869P	Probe	[6FAM]CATGCGCCAACTGCTTGATCATGC[BHQ1]	200nM		

The primers/probe set were checked for homodimer, heterodimer and hairpin using the oligoanalyzer website (https://www.idtdna.com/calc/analyzer). The presence of heterodimers was checked among *fHbp* primers and probe but also between *fHbp* and *gdh* systems.

Based on this analysis *fHbp* primers and probes do not show any homodimer, heterodimer or hairpin which may affect the efficiency of the qPCR.

Finally the sets were check for specificity using BLAST analysis against *Neisseria meningitidis* genomes and the all database (figure 1) in NCBI GenBank website.

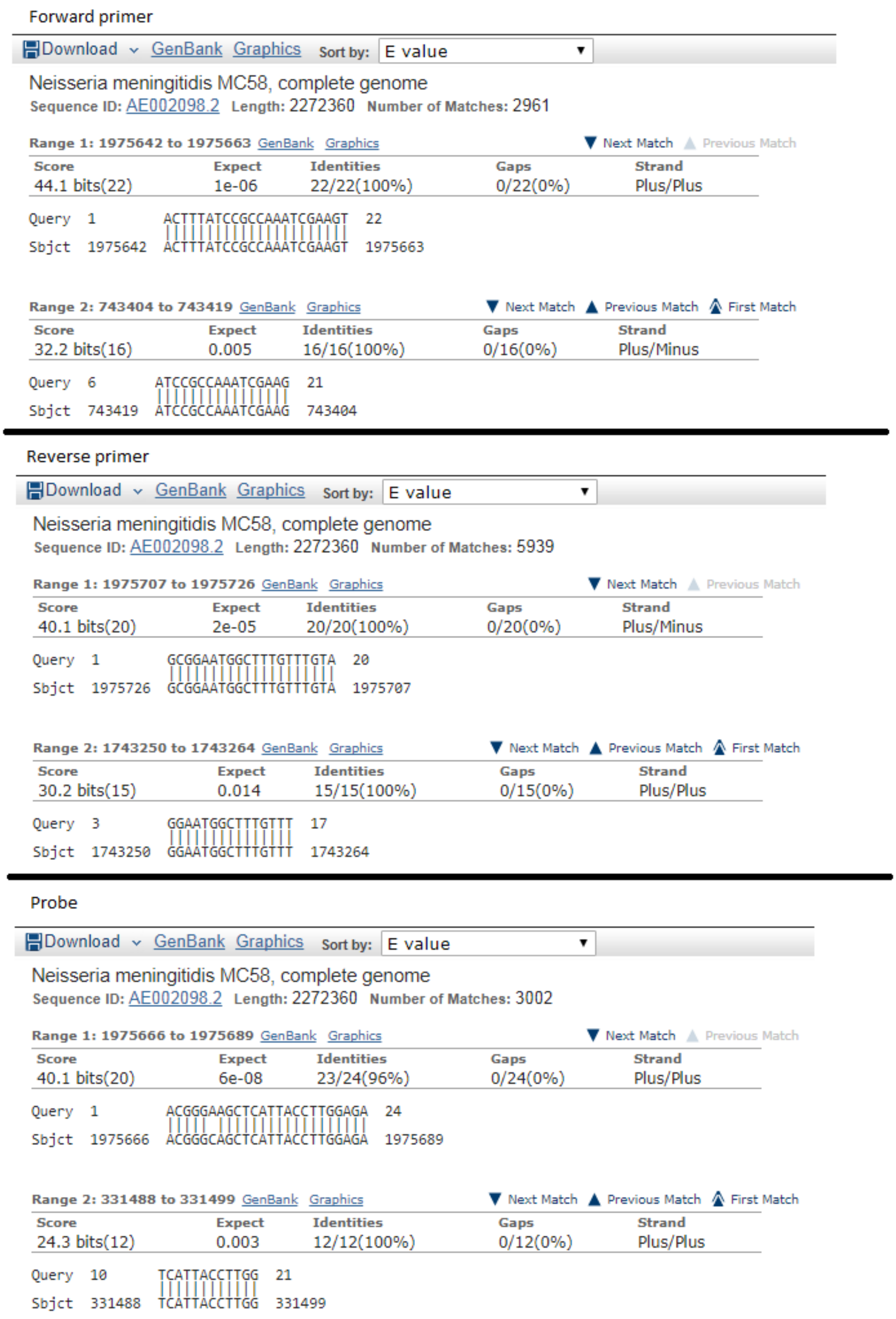

Figure 1. Example of BLAST results obtained when comparing qPCR primer against Neisseria meningitidis genome.

The BLAST results showed that *fHbp* primers and probe will bind only the *fHbp* gene as no other region showed similarities that were high enough to allow efficient amplification.

<u>Primer optimisation</u>

A gradient PCR was performed to determine the optimal melting temperatures. This gradient PCR was also used to check for the presence of secondary products.

The gradient PCR was performed as following: 1X Fast SYBRgreen master mix, 400nM primer concentration, 1ul of MC58 DNA. Amplification were performed in a Mastercycler Pro thermocycler (Eppendorf, UK) with the following parameters: 95°C for 20seconds, and 40 cycles of 95°C for 3seconds and a gradient of temperature between 56°C and 64°C for 30 seconds. PCR product were run on a 2.5% agarose gel (1X TAE buffer*)* at 100V.

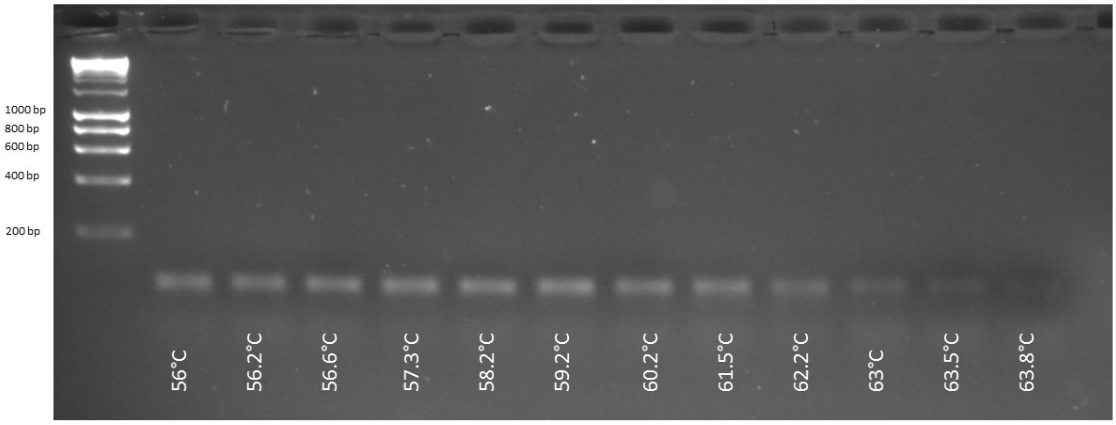

Figure 2. Gradient PCR results. First lane Hypperladder I (Bioline, UK) size marker.

Based on the gradient PCR the Tm chosen for the assay was 58°C. No secondary product were observed during the gradient PCR.

The optimal primer concentration was determined by qRT-PCR in reactions (10 µL) containing 1X Fast SYBRgreen master mix, three different primer concentration combinations (200nM/200nM, 300nM/300nM and 400nM/400nM) and 0.5 µL of a 4 log serial dilution of MC58 DNA. Amplification was performed in a 7500 Fast Applied Biosystems system with the following thermocycling parameters:- 95°C for 20 seconds; 40 cycles of 95°C for three seconds and 60°C for 30 seconds; melt curve analysis consisting of denaturation at 95°C, renaturation at 60°C for 1 minute and progressive denaturation starting at 60°C and rising by 1% to a final temperature of 95°C. The primer concentration 400nM showed the best efficiency with the lower Ct values (figure 4).

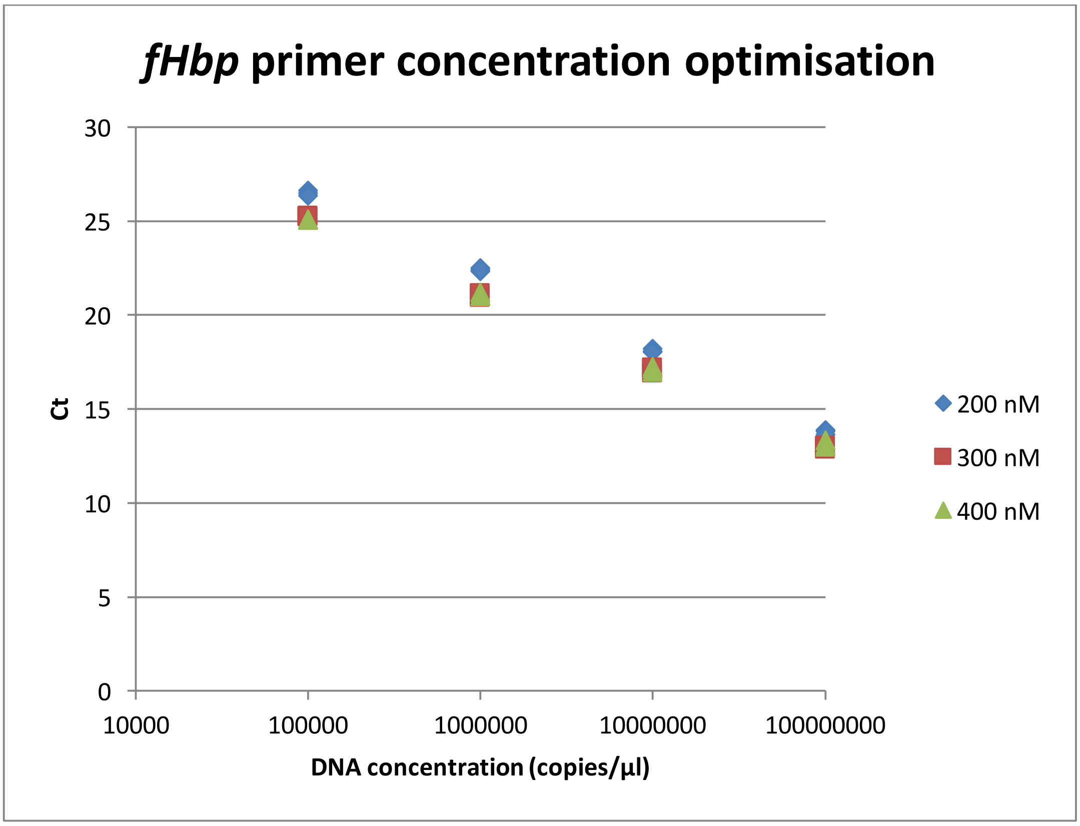

Figure 3. Primer concentration optimization results.

Once the optimal primer concentration had been determined, the optimal concentration of the hydrolysis probes was tested. The qPCR were performed as following: Reaction mixtures (10 µL) contained 0.5 µL of a MC58 DNA 4 log serial dilution, 1X TaqMan fast advance master mix (Applied Biosystems, UK), 400nM of primers, and either 200, 250, 300 nM of probes.

The qPCR assay was performed in an Applied Biosystems 7500 Fast Machine using the following conditions:- Uracil N-Glycosylase decontamination at 50°C for 2 minutes; polymerase activation at 95°C for 20 seconds; 40 cycles of denaturation at 95°C for 2 seconds and annealing/extension at 58°C (fhbp) for 30 seconds.

The probes concentration giving the best efficiency with the lower Ct value was 200nM (figure 4).

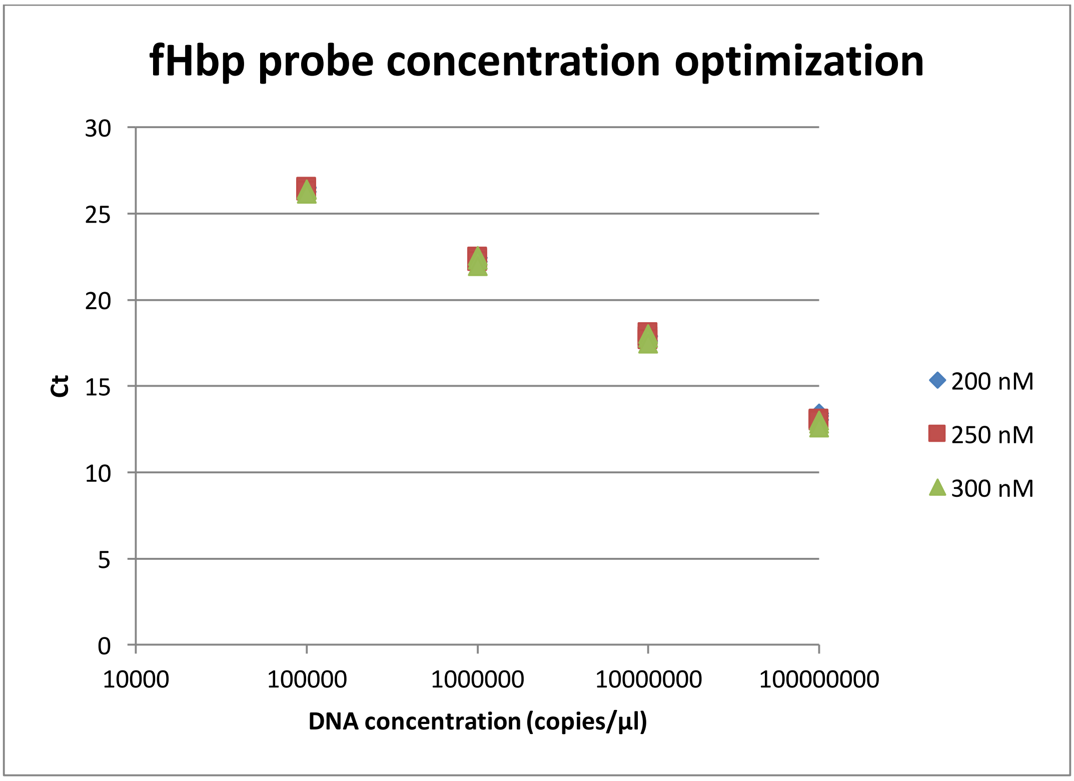

Figure 4. Probes concentration optimization results.

<u>Reference gene validation.</u>

Previously two reference genes were used for gene expression analysis by qPCR: 16S RNA ribosomal gene and *gdh* (Sanders et al. 2012; Echenique-Rivera et al. 2011).

It was decided to test those reference genes and the target genes on a small range of 11 isolates representative of the diversity of the *Neisseria meningitidis* population selected for the study (Table 2). Three mL of mid-log phase bacterial cultures (OD600nm of 0.5) were mixed immediately with 600 µL of 95% ethanol/5% phenol and incubated for 30 minutes at +4°C. After incubation the bacteria were pelleted by centrifugation and the pellet was stored at -80°C. Total RNA was extracted using a Total RNA extraction kit (Norgen, UK) as follows:- addition of 100 µL of Lysozyme solution (3 mg/mL); incubation at 37°C for 10 minutes; addition of 300 µL of lysis solution with 10 µL of β-mercaptoethanol; RNA extraction following the manufacturer's instructions; and elution in 50 µL of elution buffer. Genomic DNA was removed by two cycles of 20 minutes treatment using 0.5µl of DNase solution from the Turbo DNase kit (Ambion, UK). DNase inactivation was performed following the manufacturer instruction. After the Dnase treatment the RNA was re-purified using the Norgen extraction Kit and the RNA was eluted with 50ul of water. The concentration and the quality of the RNA was checked. A 500 ng sample of total RNA was subject to reverse transcription using the RNA to cDNA kit (Applied Biosystems, UK) following the manufacturer's instructions. All qPCR reactions were performed in their optimal conditions. For the 16S rRNA system the cDNA was diluted 1/10,000 to obtained a range of Ct comparable to the one obtained for the other genes.

We observed that 16S and *gdh* gene showed very stable Ct values (~less than 2 Ct difference) with a comparable pattern of variation (figure 5). The expression of *cbbA* was shown to be stable. As expected *fHbp* Ct values were very variable. This test confirmed that the 16S rRNA and *gdh* gene are expressed at a constant level among the tested bacterial populations.

Table 2. Sample tested for the reference gene validation.

Isolates	ST type	Clonal complex	Serogroup
M10 240701	213	213	B
M02 241729	11	11	B
M12 240202	11	11	C
M11 240077	269	269	B
M13 240048	1161	269	B
M11 240165	14	174	Y
M12 240203	1	162	B
M14 240368	ND	ND	B
M13 240134	829	198	NG
MC58	74	32	B
H44/76	32	32	B

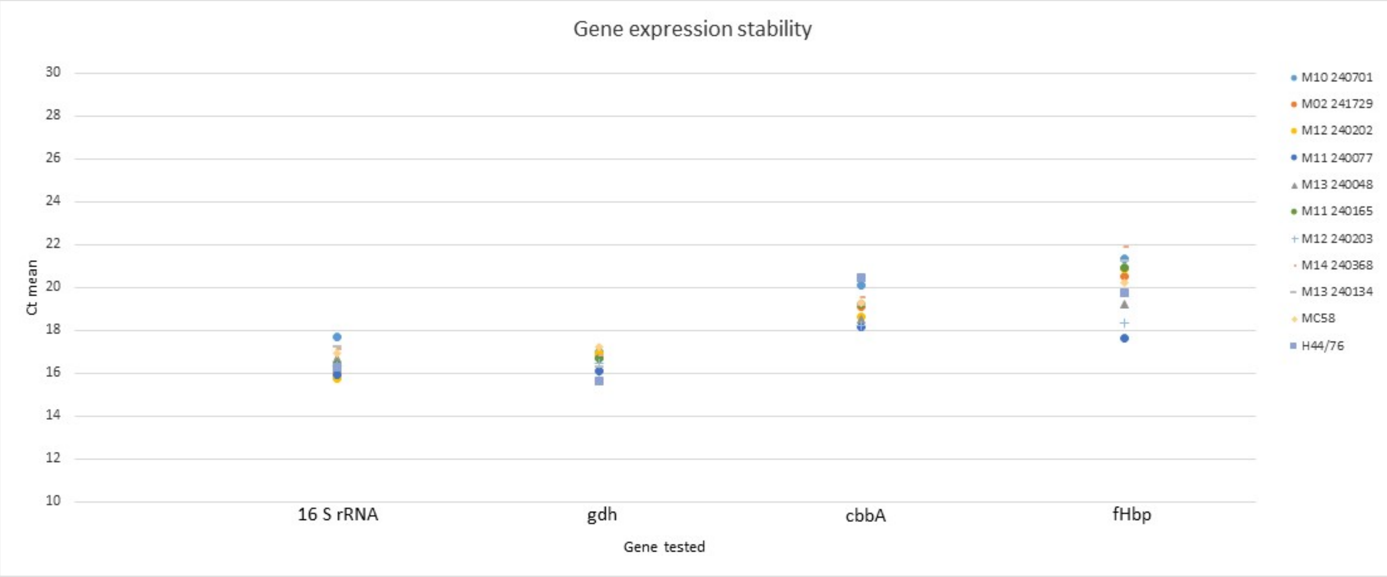

Figure 5. Gene expression stability among 11 isolates. Four genes were tested: gdh, 16S rRNA gene, fHbp and cbbA.

The 16S rRNA gene expression, due to its very high level, is difficult to compare with our target genes. For this reason, we decided not to use 16S as a reference gene

It is advised to use at least two reference genes for gene expression studies. However, the usage of several reference genes is required because the treatment and the variable conditions applied to the sample or the different types of tissue may affect some of the reference genes. In the present study the gene expression analysis was performed on *Neisseria meningitidis* isolates cultured in the exact same conditions. In this case the possibility of variation in level of expression between samples are reduced as observed in the small scale test done. For this reason, the normalization of the target genes was done by using *gdh* only.

Simplex/duplex optimisation

The efficiency and accuracy of the multiplex reaction was tested compared to the simplex reactions. For this purpose simplex and multiplex reactions were performed in parallel on four samples. The duplex was validated as both *fHbp* and *gdh* systems showed insignificant Ct variation in duplex compared to the simplex reactions (Table 3).

Table 3. Multiplex validation:

		Ct simplex	Ct duplex	Ct difference	3% error from Ct simplex
fHbp	M01 240007 cDNA	19.08	19.25	0.17	0.57
	M01 241271 cDNA	20.78	20.89	0.11	0.62
	M01 241601 cDNA	20.46	20.37	-0.08	0.61
	M02 240039 cDNA	17.42	17.54	0.12	0.52
gdh	M01 240007 cDNA	19.28	19.00	-0.28	0.58
	M01 241271 cDNA	19.20	19.26	0.06	0.58
	M01 241601 cDNA	19.61	19.85	0.25	0.59
	M02 240039 cDNA	18.73	18.34	-0.39	0.56

Method of relative quantification validation: ddCT

As all systems exhibited similar efficiency close to 100%, the possibility of using the ddCt methods to determine level of expression was explored.

A 6 log serial dilution (1:10) of cDNA was prepared and amplified by qPCR as previously described. The delta Ct means were plotted against the log of input (dilution factor of cDNA) and a linear best fit line was determined. A linear best fit line slope of 0.0824 was obtained, which is inferior to 0.1 (Figure 6). This result confirms that the ddCT methods can be used for the relative quantification of both transcripts.

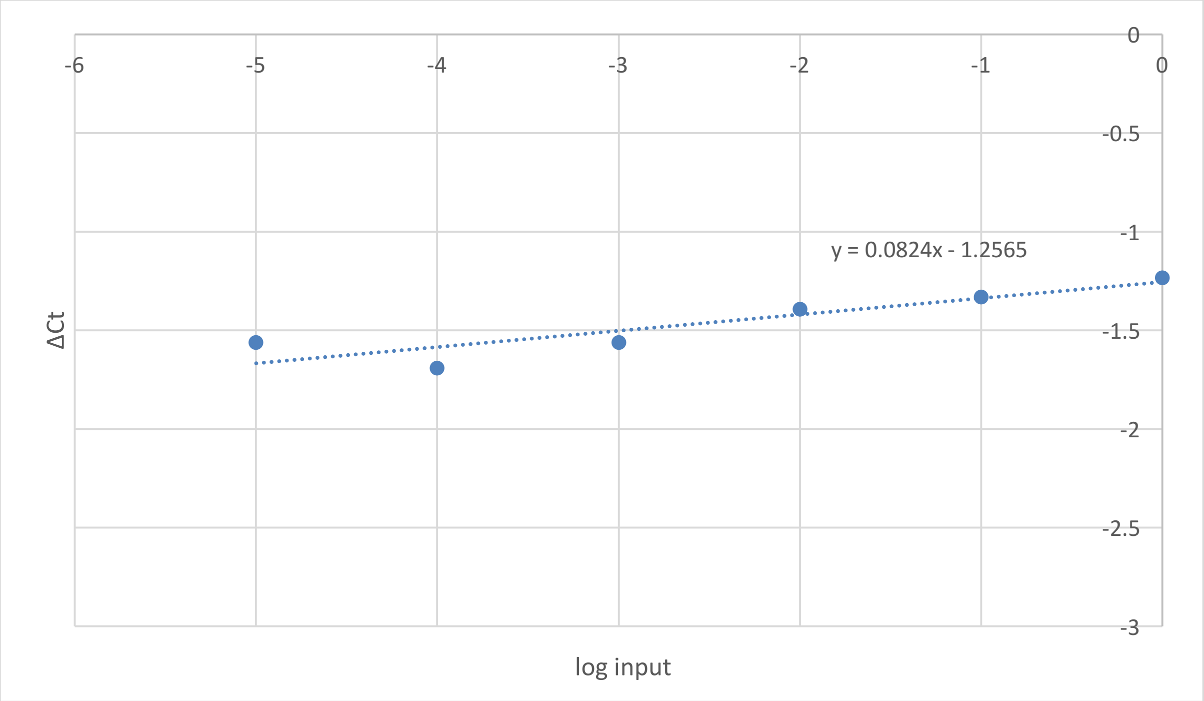

Figure 6. Comparison of the deltaCT and log input for fHbp Rt qPCR.

<u>Limit of detection and linear range test</u>

The sensitivity of *fhbp* RT qPCR assay was determined as following. A PCR ampliflying fHbp gene was performed (Lucidarme et al 2010). The concentration in DNA was estimated by nanodrop. Based on the DNA concentration the target number of copies was estimated. A serial dilution 1:10 of the target was then performed from 10^8 copies/ul to 10 copies/ul. The fHbp assay were performed as previously described.

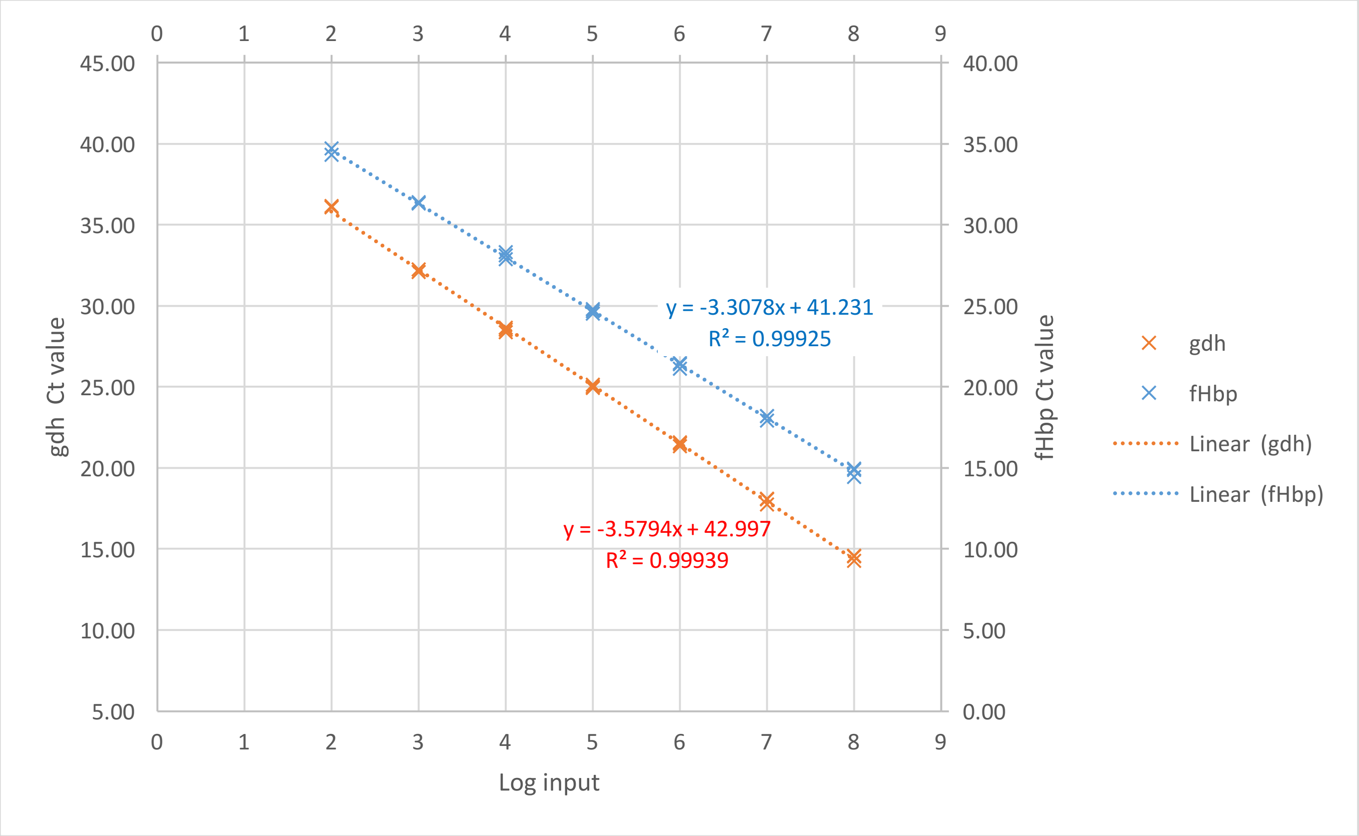

Figure 7. RT qPCR sensitivity assay.

Based on the standard curve, the lower limit of detection is 10^3 copies/µl (figure 7). In lower concentrations, some amplification was obtained but a high standard deviation between replicates was observed and some replicates were negative.

RNA extracts validation

Quantification and quality control.

After RNA extraction, RNA yield and purity were determined using the nanodrop 2000C spectrophotometer (Thermo scientific, UK). (Table 2). The RNA yield was comprised between 107.3 ng/ul and 1184.8 ng/ul.

Sample	RNA concentration (ng/µl)	260/280 ratio
M04 240731	589.4	2.05
M10 240569	243.1	1.96
M10 240579	344.3	1.84
M11 240002	322.4	2
M11 240165	313.1	1.99
M11 240389	222.9	1.99
M11 240723	290.8	2
M11 240946	274.4	2
M12 240336	329.3	1.99
M13 240134	525.8	2.07

M13 240614	397.1	1.99
M13 240675	433.3	2.02
M14 240368	420.6	2.01
M15 240147	540.9	2
M04 241215	1115.8	2.04
M11 240149	305.4	2.01
M11 241044	377.4	1.99
M12 240741	351.2	2
M13 240525	573.7	2.07
M14 240477	296.9	2.04
M11 240236	281.5	2
M12 240006	432.3	1.99
M13 240048	622.3	2.08
M13 240490	743.1	2.06
M14 240465	901.8	2.06
M01 240007	372.8	2.03
M01 240601	426.5	2.02
M02 240210	675.4	2.03
M03 240823	717	2.05
M11 240077	394.8	1.98
M11 241033	1184.8	2
M12 240831	107.3	1.98
M13 240486	990.3	2.06
M14 240434	264.5	2.05
M02 241729	639.8	1.8
M10 240684	660.8	2.03
M10 240701	231.1	1.98
M11 240108	860	2.04
M11 240247	395.8	1.99
M11 240994	388	1.99
M11 241039	854.2	1.97
M12 240290	614	1.97
M12 240698	397.8	1.97
M13 240090	376.7	1.97
M13 240254	261.6	2.13
M13 240402	576	2.07
M14 240008	417.5	2.03
M14 240094	427.4	2.01
M14 240383	393.2	2.01
M14 240476	234.9	2.04
M98 252111	553.7	2
H44/76	917.7	2.05
M11 240175	288.4	2
M12 240203	431.4	1.97

M13 240017	792.3	2.06
M14 240014	476.1	1.98
M14 240480	465.4	2.06
MC58	507.5	2.03
M01 241601	254.2	2.05
M10 240750	254.7	1.99
M11 240766	533.9	2.02
M12 240656	571.2	2.06
M14 240018	542.8	2.08
M14 240472	550.3	2.08
M11 240409	263.4	2
M12 240201	272.2	2
M13 240072	359.3	1.99
M13 240245	410.4	2.01
M14 240367	446.9	2
M11 241043	851.7	2
M12 240202	745.2	2.04
M12 240679	653.8	2.05
M13 240189	593.8	2.07
M13 240559	369.3	2.02
M11 241066	809.2	1.99
M12 240846	508.6	2.04
M13 240210	230.3	2.04
M13 240237	519.2	2.04
M13 240479	986.4	2.07

Table 2. Total RNA concentration and purity.

A total of 500ng of RNAs extract was subject to electrophoresis in 1.2% agarose gels (0.2 µg/mL ethidium bromide, 1x MOPS, 1.1% formaldehyde) to determine level of degradation. Any samples with RNA showing advance degradation (absence of rRNA bands or important degradation in the small sizes) were processed again (Figure 8).

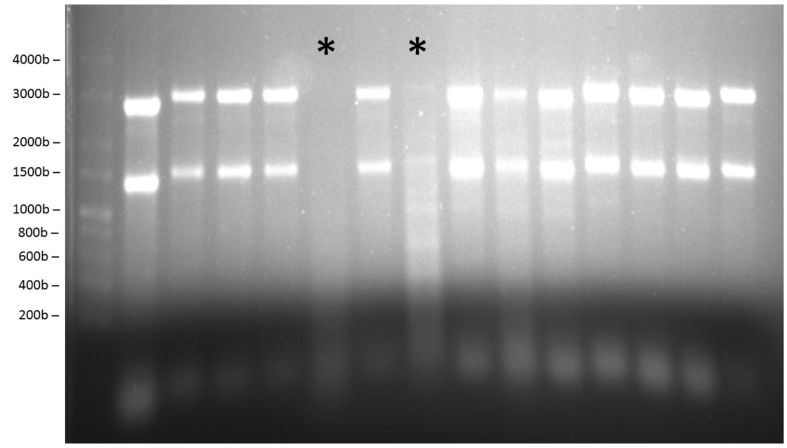

Figure 8. Gel electrophoresis of 500ng of total RNA extract. The gel show examples of validated RNA extracts and rejected RNA extracts (asterix).

All RNA extracts were tested for DNA contamination before reverse transcription. A qPCR assay targeting gdh was performed using similar conditions to the cDNA assay. Any RNA extracts exhibiting a Ct value inferior to 35 in triplicate were DNase treated again. Quality and quantity analyses were then performed again.

<u>Inhibition assay.</u>

To determine the presence of qPCR inhibitors. A 6-log serial dilution of one isolate cDNA (prepared as previously described) was performed and amplified by qPCR using fHbp and gdh duplex reaction (see above). The Ct mean obtained were plotted against log input. The curve obtained was perfectly linear with a R^2 for *fHbp* and *gdh* of 0.9994 and 0.9998 respectively. This results showed that no PCR inhibitor are present in the sample cDNA. As all the samples are cultured and extracted in the exact same conditions, we conclude that the cDNA samples do not contain PCR inhibitors.

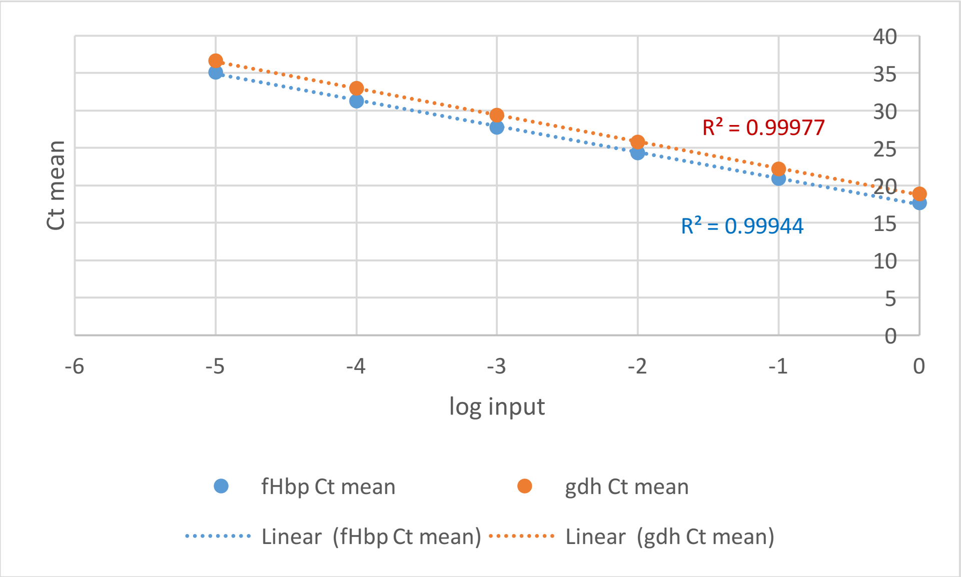

Figure 9. Inhibition assay. Log input correspond to the log of the dilution as the pure cDNA was arbitrary put at 1.

REFERENCE:

- Echenique-Rivera H, Muzzi A, Del Tordello E, Seib KL, Francois P, Rappuoli R, Pizza M, Serruto D. Transcriptome analysis of Neisseria meningitidis in human whole blood and mutagenesis studies identify virulence factors involved in blood survival. PLoS Pathog. 2011 May;7(5):e1002027
- Lucidarme J, Tan L, Exley RM, Findlow J, Borrow R, Tang CM. Characterization of Neisseria meningitidis isolates that do not express the virulence factor and vaccine antigen factor H binding protein. Clin Vaccine Immunol. 2011 Jun;18(6):1002-14.
- Sanders H, Brehony C, Maiden MC, Vipond C, Feavers IM. The effect of iron availability on transcription of the Neisseria meningitidis fHbp gene varies among clonal complexes. Microbiology. 2012 Apr;158(Pt 4):869-76
