# Supplementary material for: Clustered intergenic region sequences as predictors of factor H Binding Protein expression patterns and for assessing Neisseria meningitidis strain coverage by meningococcal vaccines
Source: PLoS One. 2018 May 30;13(5):e0197186. doi: 10.1371/journal.pone.0197186 (PMC5976157; doi:10.1371/journal.pone.0197186)
Supplement: S2 Data Set — (PDF) [file pone.0197186.s017.pdf]

Mathematical Modelling of the Agglomerative Clustering of fHbp Expression Data

Data Description

The data contains the expression measurement (*fhbp* RQ values) for 79 isolates. This expression has been classified based on the IGR present in the isolate. The scatter plot of the data is shown in figure 1 and the distribution of the IGRs is shown in table 1.

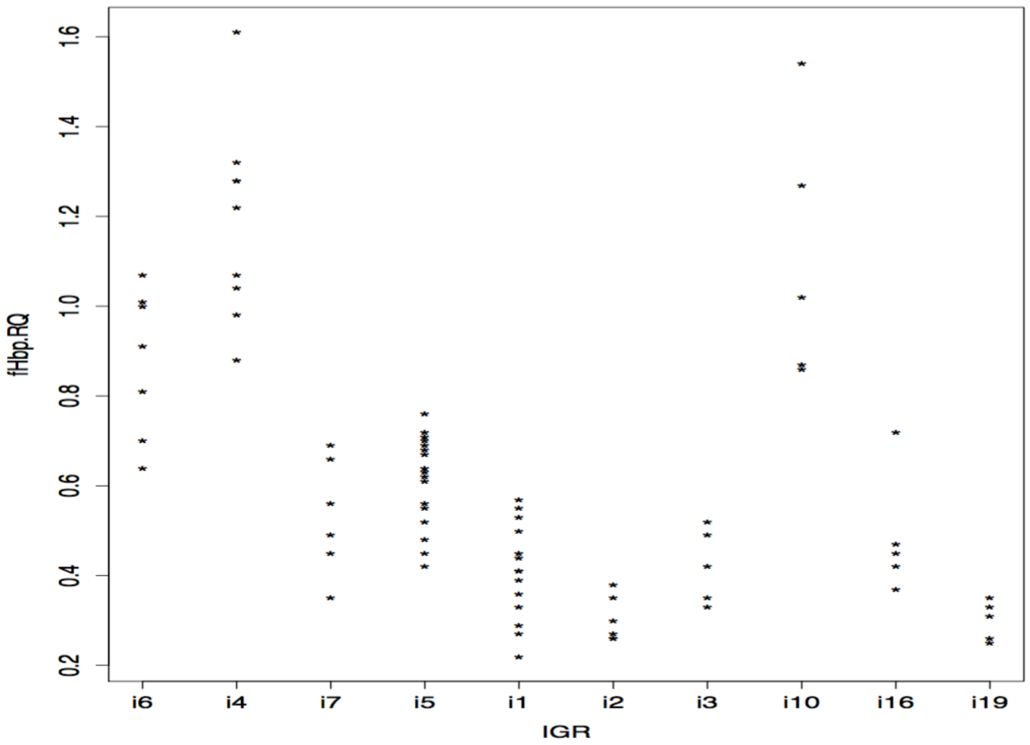

Figure 1: Scatter plot of the expression measurement (fHbp RQ)

IGR Alelles	Number of Isolates
i1	14
i2	6
i3	5
i4	9
i5	17
i6	7
i7	6
i10	5
i16	5
i19	5
**Grand Total**	**79**

Table 1: This table shows the distribution of expression measurements (fHbp RQ) by IGR

Problem Statement

In the first part of the analysis, we would like to find clusters of IGRs such that the following are satisfied:

. 1) The IGRs which belong to a cluster have mean differences which are not statistically significant.

. 2) The IGRs in different clusters have mean differences which are statistically significant.

Proposed Solution

We propose to solve this problem by using hierarchical clustering with similarity defined in the

sense of statistical significance of difference between the mean expression values for each IGR.

Algorithm

The approach used to generate this cluster is given below:

. a) We apply a *t* test to each pair of IGRs in the data to assess the significance of differences in mean expression values (see table 2 for an example).

. b) For each IGR, we find all other IGRs with means which are statistically significant at a significance level of 0.05 (see table 3 for an example)

. c) We find the pair of IGRs with means which are ***most similar***. Similarity $S\,(A, B)$ between 2 IGRs A and B here is defined as $S(A,B) = [1 - pvalue(A,B)]$ where $pvalue(A, B)$ is the p value of the *t*-test between A and B. The closer $S\,(A, B)$ is to zero the more similar A and B. To find the ***most similar*** IGRs, we find $S^{*}(A, B) = \min_{A,B \in IGR} [1 - pvalue(A, B)$. That is we find the pair of IGRs with the highest p value as compared to all other pairs of IGRs. If this is not unique, we randomly select one of them.

. d) If the *p value* between the most similar IGRs found in *step c* is not statistically significant (i.e. *p value* > 0.05), then we merge this pair of IGRs and repeat *step a – d*.

The algorithm described above is a form of hierarchical clustering with a similarity measure defined as 1-p value of a t-test.

The next two tables show the results obtained from the first iteration of the algorithm.

IGR	i6	i4	i7	i5	i1	i2	i3	i10	i16	i19
**i6**	-	0.0062	0.0015	0.0043	0.0001	0.0000	0.0001	0.1561	0.0013	0.0000
**i4**	0.0062	-	0.0000	0.0000	0.0000	0.0000	0.0000	0.6334	0.0000	0.0000
**i7**	0.0015	0.0000	-	0.2152	0.0711	0.0057	0.1211	0.0081	0.5724	0.0054
**i5**	0.0043	0.0000	0.2152	-	0.0000	0.0000	0.0029	0.0172	0.1082	0.0000
**i1**	0.0001	0.0000	0.0711	0.0000	-	0.0083	0.7818	0.0047	0.2945	0.0063
**i2**	0.0000	0.0000	0.0057	0.0000	0.0083	-	0.0302	0.0030	0.0368	0.9094

**i3**	0.0001	0.0000	0.1211	0.0029	0.7818	0.0302	-	0.0046	0.4015	0.0273
**i10**	0.1561	0.6334	0.0081	0.0172	0.0047	0.0030	0.0046	-	0.0055	0.0030
**i16**	0.0013	0.0000	0.5724	0.1082	0.2945	0.0368	0.4015	0.0055	-	0.0350
**i19**	0.0000	0.0000	0.0054	0.0000	0.0063	0.9094	0.0273	0.0030	0.0350	-

Table 2: This table shows the *p* value of pairwise t tests. For this test, the *p* value is the probability of observing by chance the same or greater difference of means if two samples are taken from populations with the same mean. The cells coloured in blue are those with no statistical significance. -, no data as IGRs are not compared against themselves.

**IGR**	**i6**	**i4**	**i7**	**i5**	**i1**	**i2**	**i3**	**i10**	**i16**	**i19**
**i6**	0	1	1	1	1	1	1	0	1	1
**i4**	1	0	1	1	1	1	1	0	1	1
**i7**	1	1	0	0	0	1	0	1	0	1
**i5**	1	1	0	0	1	1	1	1	0	1
**i1**	1	1	0	1	0	1	0	1	0	1
**i2**	1	1	1	1	1	0	1	1	1	0
**i3**	1	1	0	1	0	1	0	1	0	1
**i10**	0	0	1	1	1	1	1	0	1	1
**i16**	1	1	0	0	0	1	0	1	0	1
**i19**	1	1	1	1	1	0	1	1	1	0

Table 3: In this table value 1 is used to denote a pair of IGRs with mean differences which are statistically significance while cells with value 0 are for pairs of IGRs with mean differences which are not statistically significant.

The result generated from the algorithm is shown below in table 4.

**Cluster (Name)**	**IGR Merged**

Cluster 1 (i20)	i2 and i19
Cluster 2 (i21)	i1 and i3
Cluster 3 (i22)	i4 and i10
Cluster 4 (i23)	i7 and i16
Cluster 5 (i5)	i5
Cluster 6 (i6)	i6

Table 4. Definition of clusters.

The scatter plot of the clusters is shown below in figure 2.

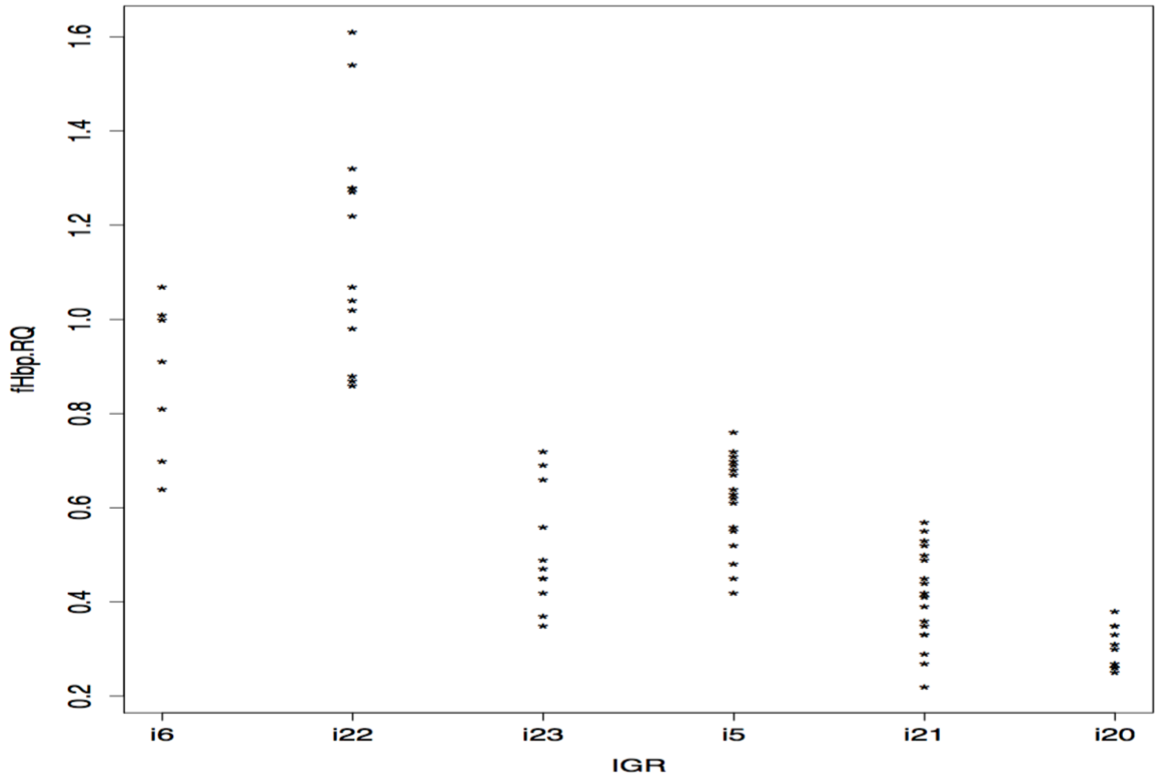

Figure 2: Scatter plot of the observations by cluster. See table 4 for the cluster definition.

Conclusion

In conclusion, we were able to identify 6 clusters of IGRs such that within each cluster the

differences in the mean expression values of the member IGRs are not significantly different from each other whereas the differences between the means of each group are statistically significant.
