# Supplementary material for: Clustered intergenic region sequences as predictors of factor H Binding Protein expression patterns and for assessing Neisseria meningitidis strain coverage by meningococcal vaccines
Source: PLoS One. 2018 May 30;13(5):e0197186. doi: 10.1371/journal.pone.0197186 (PMC5976157; doi:10.1371/journal.pone.0197186)
Supplement: S3 Data Set — (PDF) [file pone.0197186.s018.pdf]

Report 5

This report contains the results of the analyses of the RQ data for the cbba gene. This analyses are similar to the one done for fHbp in previous reports.

Data Description

The first file contains the RQ data. There are 79 samples together with their IGR groups. See chart 1 for the distribution of the data. The second data file contains the intergenic region sequences containing the promoters. This contains 6 sequences.

Chart 1: Initial Distribution of the cbba_RQ Data

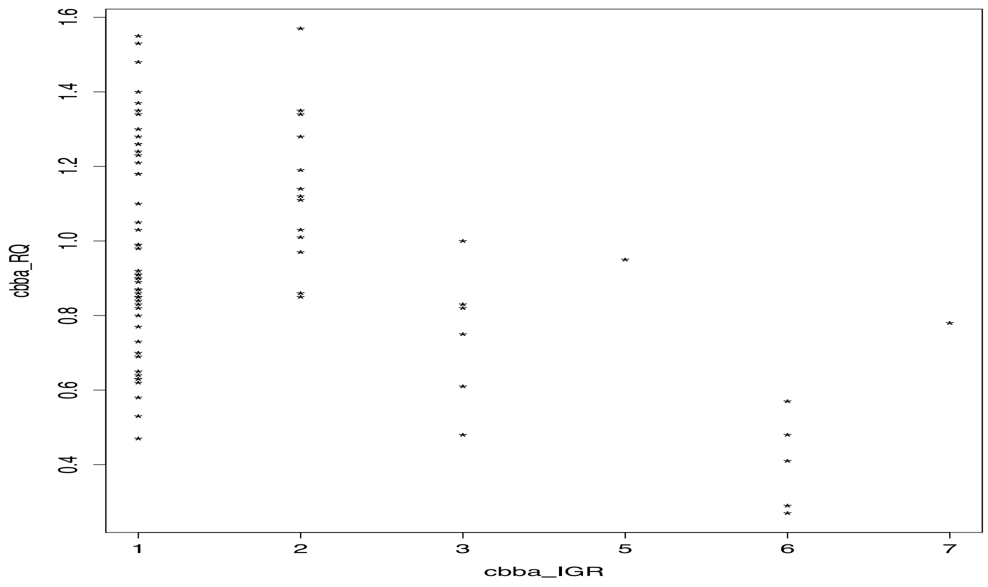

Task 1

The first task is to find the sequences of IGR which are associated with certain level of expression. To do this we find clusters of IGRs such that the following are satisfied:

1) The cbba_RQ expression for the IGRs which belong to a cluster have differences of means which are not statistically significant.
2) The average of the cbba_RQ expressions in different clusters are statistically significantly different.

For each pair of IGR groups, we tested the null hypothesis that the means of their cbba_RQ are equal. We define the similarity between a pair of IGR groups as the *p*-value of the *t*-test on their respective cbba_RQ. We select 95% as level of significance. IGR group 5 and 7 contains one sequence each (see chart 1). For *t*-test on a pair which include IGR groups 5 (or group 7), we tested the null hypothesis that the mean of the group is equal to the value of cbba_RQ in group 5 (or group 7) and define the similarity as the *p*-value of this test. The pair of IGR groups which contain both group 5 and 7, was exempted since there is no statistic to test.

We applied agglomerative clustering to merge similar IGR groups, defining the similarity of groups as given above. Chart 2 shows the distribution of the cbba_RQ before clustering (same as chart 1 with the quartile added) and after clustering.

Chart 2: Distribution of cbba_RQ for each IGR group before clustering

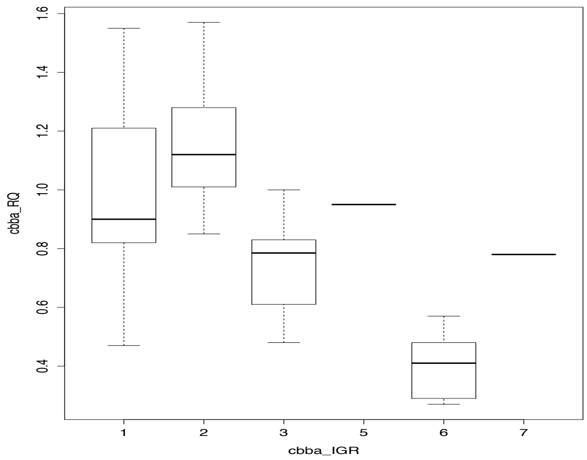

Figure A: *Distribution of cbba_RQ for each IGR group before clustering*

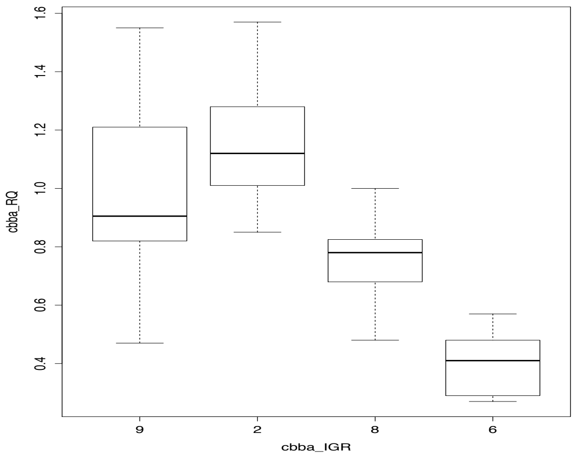

Figure B: *Distribution of cbba_RQ for each IGR group after clustering*

See table 1 below showing the details of the merged groups. The details of the *t*-test are given in the appendix.

Table 1:

**Cluster Name**	**IGR Merged**
Cluster 9	IGRs 1 and 5
Cluster 2	IGR 2
Cluster 8	IGRs 3 and 7
Cluster 6	IGR 6

From the result given in table 1, we observed that the single points in group 5 was absorbed into group 1 while the single point in group 7 was absorbed into grouped 3. Apart from the absorbed points, the means of all the remaining groups were statistically significantly different at 95% confidence level.

Task 2

We have 6 IGR sequences. After removing the non-variable positions, we have only 6 positions remaining. Each sequence has a different IGR label. For each IGR sequence we have the average cbba_RQ which was set as the target variable. The task here is to induce a model which can predict the average cbba_RQ that will be expressed based on the sequences and to identify the IGR position which affects the level of cbba_RQ produced.

Data Pre-Processing

For the sequences, most of the positions (attribute) are binary valued (and were coded by 0 and 1) except the position 5 which has three values and was coded using 2 binary variables V5_a and V5_b.
Therefore, the data matrix for analysis consists of 6 rows (sequences), 7 positions (columns /variables). The column vector with average cbba_RQ expression is called target. See table 2 for full data matrix. The details of the codes are provided in the appendix.

Table 2

Sequence Number	**V1**	**V2**	**V3**	**V4**	**V5_a**	**V5_b**	**V6**	**Average cbba_RQ**
Sequence 1	0	0	0	0	0	0	0	0.9715
Sequence 2	0	0	0	0	1	0	0	1.1400
Sequence 3	0	0	0	0	0	0	1	0.4040
Sequence 4	0	1	0	0	0	1	0	0.9500
Sequence 5	0	1	1	1	0	1	0	0.7483
Sequence 6	1	1	1	1	0	1	0	0.7800

***Note**: that the columns V5_a and V5_b are the result of coding the ternary position 5 as two binary variables.*

Analysis

Our data is binary valued whereas our target is continuous. Therefore, we create a simple regression model. This can be used to understand the relationship between the sequence positions and the cbba expression levels, and also for prediction of cbba_RQ expression level.

We observe co-segregation between variant positions (See table 2). For example, V2 and V5_a are linked (we merge V5_b into V2), also V3 and V4 are co-segregating (we merge V3 and V4 to form V3/V4). The final data matrix for analysis is shown in table 3 below.

Table 3

Sequence Number	**V1**	**V2**	**V3/V4**	**V5_a**	**V6**	**IGR**	**Average cbba_RQ**
Sequence 1	0	0	0	0	0	IGR 1	0.9715
Sequence 2	0	0	0	1	0	IGR 2	1.1400
Sequence 3	0	0	0	0	1	IGR 6	0.4040
Sequence 4	0	1	0	0	0	IGR 5	0.9500
Sequence 5	0	1	1	0	0	IGR 3	0.7483
Sequence 6	1	1	1	0	0	IGR 7	0.7800

Regression Analysis

We created a linear regression using the variables in table 3 to predict the average cbba_RQ expression. Due to the small sample size (6) compared to the number of variable (5), we carry out further variable selection. We randomly removed a variable from the data and then create a linear regression model, the removed variable is then re-introduced back while the worst performing variable is removed and model re-created. The worst performing variable for removal is the non statistically significant variable with the largest *p*-value. This analysis was repeated using all the variable until we have a minimal subset of variables whose coefficients were statistically different from zero. The final result gives 3 variables which are positions V3/V4, V5_a, V6. The details of the regression model are given in the appendix.

Results and Discussion

The prediction of the group average cbba_RQ expression level based on these 3 variables has a goodness of fit of 99% (the percentage of explained variance).
Usage of these variables also identifies the cluster which was found by the application of *t*-test in task 1. See Table 4.

Table 4

Sequence Number	**V3/V4**	**V5_a**	**V6**	**IGR**	**Cluster**
Sequence 1	0	0	0	IGR 1	Cluster 9
Sequence 2	0	1	0	IGR 2	Cluster 2
Sequence 3	0	0	1	IGR 6	Cluster 6
Sequence 4	0	0	0	IGR 5	Cluster 9
Sequence 5	1	0	0	IGR 3	Cluster 8
Sequence 6	1	0	0	IGR 7	Cluster 8

Cluster defined by regression variables.

Table 5:

**Cluster (Name)**	**IGR Merged by *t*-test**	**IGR Merged by linear regression**
Cluster 9 (Ecb2)	Clusters 1 and 5	Clusters 1 and 5
Cluster 2 (Ecb1)	Cluster 2	Cluster 2
Cluster 8 (Ecb3)	Clusters 3 and 7	Clusters 3 and 7
Cluster 6 (Ecb4)	Cluster 6	Cluster 6

Note: Shows the comparison of the cluster defined by regression variable to those defined using agglomerative clustering using *t*-test as a measure of similarity (as earlier defined); *cbba* expression clusters (Ecb) were named and numbered from high (1) to low (4) expression

We have few sample points for analysis and therefore the statistical power is not much, but if we can find cluster by 2 different methods then we may conclude that we have some evidence to accept clusters.

The 3 attributes resulting from the regression analysis can be taken as variables which influences the level of cbba_RQ expression.

Some Notes

We identified 3 variables which have some influence on the level of cbba_RQ expression. This requires further investigation as the data sample is few.

We observed large variation in the cbba_RQ level of some IGRs groups. This may imply that our assumption that cbba_RQ level can be completely defined by IGR may not be entirely correct. However, this hypothesis requires further investigation especially since we have few measurements for some IGR groups.

Task 3

The task here is to calculate the probability that a group of samples with the same peptide will exhibit a fHbp relative potency under the threshold given as 0.012. The distribution of the peptide is given in chart 3 below.

Chart 3

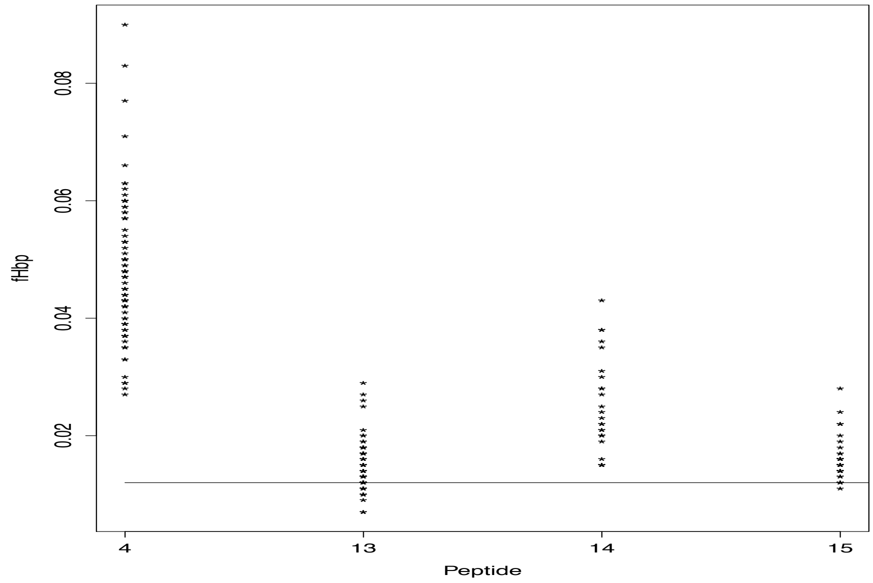

Under the assumption that the data is uniformly distributed for each of the peptide types, we estimate the probability that the peptide potency level fall below the threshold $t = 0.012$, using proportion estimation. The result and the confidence interval at 95% confidence level is given below in table 6.

Table 6

**Peptide Type**	**Probability (fHbp relative potency <0.012)**	**Confidence Interval**	
		Lower limit	***Upper limit***
4	0	0	0.0406
13	0.1818	0.0976	0.2961
14	0	0	0.1322
15	0.0313	0.0008	0.1622

However, if the data are not uniformly distributed and we can replace the assumption that the data are uniformly distributed with the assumption that the data are normally distributed. This is more suitable for estimating the probability that a given peptide falls below the threshold t especially for cases where the sample does not contain any case that falls below the threshold such as 4 and 14. See table 7 for the result.

Table 7

**Peptide Type**	**Probability (fHbp relative potency <0.012)**	**Parameters**	
		Sample mean	***Sample variance***
4	0.009	0.0480	0.0001
13	0.2567	0.0149	1.9e-05
14	0.0543	0.0249	6.5e-05
15	0.1382	0.0161	1.4e-05

We can see that probabilities estimated under normality assumption (Table 7) are inside the 95% confidence intervals for uniformity assumption.

Appendix

Table A

Codes for Sequences

Nucleotide position	Code	
	**0**	**1**
P1	G	A
P2	C	T
P3	C	T
P4	C	T
P5_a	C, T	A
P5_b	A, C	T
P6	A	G

Table B

p-values of the *t*-test at the initial state of agglomerative clustering

IGR	*Group 1*	*Group 2*	*Group 3*	*Group 5*	*Group 6*	*Group 7*
Group 1	0	0.0216	0.0286	0.5670	2.95E-05	4.36E-06
Group 2	0.0216	0	0.0015	0.0062	8.77E-07	4.07E-05
Group 3	0.0286	0.0016	0	0.0423	0.0053	0.6883
Group 5	0.5670	0.0062	0.0423		0.001	
Group 6	2.9E-05	8.77E-07	0.0053	0.001	0	0.0027
Group 7	4.3E-06	4.07E-05	0.6883		0.0027	

Table C

Table shows the statistical significance of pairwise *t*-test (at 95% confidence level) at the initial state of agglomerative clustering

**IGR**	*Group 1*	*Group 2*	*Group 3*	*Group 5*	*Group 6*	*Group 7*
Group 1		1	1	0	1	1
Group 2	1		1	1	1	1
Group 3	1	1		1	1	0
Group 5	0	1	1		1	
Group 6	1	1	1	1		1
Group 7	1	1	0		1	

Note: *0 represent pairs of groups that have statistically insignificant differences of means while 1 represent pairs of groups that have statistically significant differences of means.*

Table D

p-values of the *t*-test at the final state of agglomerative clustering

**IGR**	*Group 9*	*Group 2*	*Group 8*	*Group 6*
Group 9	0	0.0208	0.0127	<0.0001
Group 2	0.0208	0	0.0004	<0.0001
Group 8	0.0127	0.0004	0	0.0021
Group 6	<0.0001	<0.0001	0.0021	0

Table E

Table shows the statistical significance of pairwise *t*-test at 95% confidence interval the after agglomerative clustering

**IGR**	*Group 9*	*Group 2*	*Group 8*	*Group 6*
Group 9		1	1	1
Group 2	1		1	1
Group 8	1	1		1
Group 6	1	1	1	

Note: *0 represent pairs of groups that have statistically insignificant differences of means while 1 represent pairs of groups that have statistically significant differences of means.*

Regression Analysis Model

SUMMARY

OUTPUT

Regression Statistics	
Multiple R	0.9989
R Square	0.9977
Adjusted R Square	0.9943
Standard Error	0.0191
Observations	6

ANOVA

	df	*SS*	*MS*	*F*	*Significance F*
Regression	3	0.3204	0.1068	291.5230	0.0034
Residual	2	0.0007	0.0004		
Total	5	0.3211			

	Coefficients	*Standard Error*	*t Stat*	*p-value*
Intercept	0.9608	0.0135	70.9863	0.0002
V3/V4	-0.1966	0.0191	-10.2708	0.0093
V5_a	0.1792	0.0234	7.6463	0.0167
V6	-0.5568	0.0234	-23.7501	0.0018
